# Role of mTOR Downstream Effector Signaling Molecules in *Francisella Tularensis* Internalization by Murine Macrophages

**DOI:** 10.1371/journal.pone.0083226

**Published:** 2013-12-03

**Authors:** Michael W. Edwards, James A. Aultman, Gregory Harber, Jay M. Bhatt, Elizabeth Sztul, Qingan Xu, Ping Zhang, Suzanne M. Michalek, Jannet Katz

**Affiliations:** 1 Department of Pediatric Dentistry, University of Alabama at Birmingham, Birmingham, Alabama, United States of America; 2 Department of Microbiology, University of Alabama at Birmingham, Birmingham, Alabama, United States of America; 3 Department of Cell, Developmental and Integrative Biology, University of Alabama at Birmingham, Birmingham, Alabama, United States of America; University of Louisville, United States of America

## Abstract

*Francisella tularensis* is an infectious, gram-negative, intracellular microorganism, and the cause of tularemia. Invasion of host cells by intracellular pathogens like *Francisella* is initiated by their interaction with different host cell membrane receptors and the rapid phosphorylation of different downstream signaling molecules. PI3K and Syk have been shown to be involved in *F. tularensis* host cell entry, and both of these signaling molecules are associated with the master regulator serine/threonine kinase mTOR; yet the involvement of mTOR in *F. tularensis* invasion of host cells has not been assessed. Here, we report that infection of macrophages with *F. tularensis* triggers the phosphorylation of mTOR downstream effector molecules, and that signaling via TLR2 is necessary for these events. Inhibition of mTOR or of PI3K, ERK, or p38, but not Akt signaling, downregulates the levels of phosphorylation of mTOR downstream targets, and significantly reduces the number of *F. tularensis* cells invading macrophages. Moreover, while phosphorylation of mTOR downstream effectors occurs via the PI3K pathway, it also involves PLCγ1 and Ca^2+^ signaling. Furthermore, abrogation of PLC or Ca^2+^ signaling revealed their important role in the ability of *F. tularensis* to invade host cells. Together, these findings suggest that *F. tularensis* invasion of primary macrophages utilize a myriad of host signaling pathways to ensure effective cell entry.

## Introduction

*Francisella tularensis* subspecies *tularensis* (Type A) and subspecies *holartica* (Type B) are highly infectious, Gram-negative, intracellular pathogens that cause tularemia, a disease with significant morbidity and mortality in humans and other mammals. Due to its ease of infection and means of dissemination, these *F. tularensis* subspecies are classified as select agents [1,2]. *F. tularensis* can infect a variety of host cells, but macrophages seem to be a very effective cell type for the replication and survival of this bacterium [3,4]. The *F. tularensis* Live Vaccine Strain (LVS) derived from subspecies *holartica* causes an attenuated form of infection in humans and has been used as a vaccine, although it is not licensed. Conversely, *F. tularensis* LVS infection of mice does cause a pathology that resembles that observed in humans infected with virulent *Francisella* strains. Since the intracellular life cycle of *F. tularensis* LVS is similar to that of type A *Francisella*, its use in research has been of great advantage [5]. 

Intracellular bacteria like *F. tularensis* have devised sophisticated mechanisms that exploit, trigger, and activate host signal transduction pathways for their internalization into mammalian cells. Central to the internalization of bacteria, including that of *F. tularensis*, is the rearrangement of the actin cytoskeleton [6]. Actin remodeling during bacterial infection can occur through the participation of various host cell receptors linked to downstream signaling molecules necessary for bacterial host cell entry that ultimately will be associated with the regulation of actin proteins. For instance, the phosphoinositide kinase-3 (PI3K), the tyrosine kinase Syk, and the extracellular regulated kinase (ERK) have been implicated in the internalization of *F. tularensis* [3,7], and these molecules directly interact with actin [8] or participate in actin regulation [9,10]. Downstream of the PI3K/Akt pathway is the master regulator serine/threonine kinase mammalian target of rapamycin (mTOR), which has been shown to be involved in the modulation of actin via downstream effectors of mTOR complex 1 (mTORC1) [11] and mTORC2 [12,13]. Yet, the importance of the mTOR pathway in *F. tularensis* invasion has not been assessed. Evidence suggest that phospholipases play a role in phagocytosis, e.g., phospholipase C (PLC), which is activated downstream of PI3K, has been shown to be important for FcγR-mediated phagocytosis [14] and for host cell uptake of *Escherichia coli* [15,16]. Moreover, PLCγ1 was shown to be involved in the modulation of mTOR in an Akt-independent manner [17]; however, whether the PLC pathway is associated with the regulation of mTOR downstream effector molecules and with *F. tularensis* infection is not known.

The kinase mTOR is found in all eukaryotes [18,19] and plays a major role in key aspects of cell biology, including membrane trafficking, cell growth and survival [20-22]. Studies on the involvement of mTOR in actin regulation have shown that knockdown of rictor resulted in defective actin cytoskeleton rearrangement [12,13]. Moreover, ERK and Akt signaling molecules are regulated by mTORC2, and these molecules seem to be implicated in actin regulation, as exemplified by the necessity for ERK in the cell entry of *Francisella novicida* [7] and *Chlamydia pneumoniae* [23], and for Akt in the internalization of *Pseudomonas aeruginosa* [24]. Downstream effectors of mTORC1, such as the 70 kDa ribosomal S6 kinase (p70S6K), known to be important in cell growth, have been recently reported to also regulate the actin cytoskeleton [11]. In addition, phosphorylation of its downstream effector ribosomal protein S6 was enhanced during phagocytosis in macrophages [25]. Rapamycin, a powerful and specific inhibitor of mTOR downstream signaling [18,26,27], can abrogate the mTORC1 pathway through its binding to FK506-binding protein 12 (FKBP12). The region of mTOR that binds the FKBP12-rapamycin complex is known as the FRB domain. mTOR forms a scaffold complex with other proteins that regulate different arms of the mTOR signaling cascade. The association of raptor with mTOR (mTORC1) is indispensable for mTOR signaling, as shown by RNA interference in cultured mammalian cells [28]. However, raptor does not affect the catalytic function of mTOR, but serves as a scaffold for the juxtaposition of mTOR with its substrates p70S6K and eukaryotic initiation factor 4E-binding protein 1 (4E-BP1) [29,30]. Hence, binding of the FKBP12-rapamycin complex to the mTOR FRB promotes a substantial dissociation of raptor from mTOR, thereby separating mTOR from its substrates and abrogating mTOR signaling, but not its intrinsic autophosphorylating catalytic activity [18]. Albeit rapamycin was known to affect only the mTORC1 pathway, recent studies demonstrated that although mTORC2 does not interact with FKBP12-rapamycin, the assembly of mTORC2 is inhibited by rapamycin following long-term treatment [31]. Since mTORC2 was reported to phosphorylate and activate Akt^Ser473^, rapamycin cell treatment consequently affected Akt^Ser473^ phosphorylation [31]. Akt activation occurs following extracellular agonist-induced PI3K stimulation, and phosphorylation at Thr^308^ and at Ser^473^ residues of Akt allows its full activation. Akt is critical in cellular processes like growth, differentiation and proliferation, and is believed to bridge the PI3K pathway to mTORC1 signaling [22]. Thus, while Akt is an upstream activator of mTORC1, Akt is also the target of mTOR via mTORC2. 

PLC is important in mediating signal transduction from extracellular and intracellular stimuli and in its involvement in phagocytic signaling [32-34]. PLCγ signaling can be activated downstream of PI3K through interactions between their SH2 and/or PH domains with phosphatidyl-inositol-3,4,5-triphosphate, thus linking the PI3K and the PLCγ/Ca^2+^ signaling pathways [35]. Furthermore, PLCγ1 has been shown to control the activation of the mTORC1 downstream effector molecule p70S6K in an Akt-independent manner, acting in parallel with the classical PI3K/Akt pathway [17].

The PI3K/Akt/mTOR cascade can respond to a myriad of stimuli through specific receptors such as Toll-like receptors. Activation of mammalian host cell transduction pathways is an essential step for the invasion of intracellular pathogens, including *F. tularensis. F. tularensis* is a TLR2 agonist that induces activation of the mitogen-activated protein kinases (MAPK) pathway, shown to play a role in the regulation of mTOR downstream targets [36-40]. Yet, the involvement of TLR2 signaling in *F. tularensis* cell entry in the context of the mTOR signaling cascade has not been studied. 

The aims of this study were to (i) determine the phosphorylation of specific signaling molecules associated with the mTOR signaling cascade in response to *F. tularensis* LVS infection, and (ii) to identify specific signaling molecules/pathways that play a significant role in *F. tularensis* LVS invasion. Our results suggest that the mTOR signaling cascade plays a prominent role in *F. tularensis* infection of primary macrophages, thus, augmenting our understanding of the signaling events involved in the invasion process by this bacterium. Furthermore, inhibition of mTOR and PI3K signaling affected the architecture of the actin cytoskeleton, inducing numerous thick, short filaments and small patches distributed throughout the cell, which significantly affected bacterial cell entry. Moreover, inhibition of MEK/ERK and p38 MAPK, but not Akt signaling resulted in a decrease in the phosphorylation of mTOR downstream effector molecules and of internalized *F. tularensis* in primary macrophages. Our results further show that PLC and Ca^2+^ signaling are also important for *F. tularensis* infection, since their inhibition significantly decreased the ability of the bacteria to invade the host cells, and abrogation of these signaling molecules affected the phosphorylation of mTOR downstream effectors. Overall, our findings suggest that phosphorylation of the mTOR signaling cascade via various cell signaling pathways is a critical strategy used by *F. tularensis* to ensure host cell invasion.

## Materials and Methods

### Ethics Statement

All studies were done in accordance with the recommendations of the Guide for the Care and Use of Laboratory Animals of the National Institute of Health. All protocols involving animal research were approved by the Institutional Animal Care and Use Committee of the University of Alabama at Birmingham (UAB; Protocol number 09112 under Institutional Animal Assurance Number A-3255-01).

### Bacteria

*F. tularensis* LVS (ATCC 29684; American Type Culture Collection, Rockville, MD), a gift from Karen Elkins (Food and Drug Administration, Rockville, MD), was grown and maintained as described previously [41,42]. 

### Mice and peritoneal macrophage cultures

C57BL/6 wild type (WT), TLR2 knockout (KO), TLR1KO, TLR6KO and TLR4KO mice were bred and maintained within an environmentally controlled, pathogen-free animal facility at the University of Alabama at Birmingham (UAB). The original WT mice were obtained from NCI/Frederick. The TLRKO breeding pairs, backcrossed >8 times onto the C57BL/6 background, were originally obtained under a material transfer agreement from Shizuo Akira (Osaka University, Osaka, Japan). All studies used 8-10 week old female mice, and all protocols were approved by the UAB Institutional Animal Care and Use Committee. 

Peritoneal macrophages from C57BL/6 WT or from the corresponding TLRKO mice were induced by i.p. injection of 1 ml of sterile 3% BBL Brewer modified thioglycollate medium (BD Biosciences), and were isolated as previously described [43]. Cells were washed and cultured in 24-well plates (1 x 10^6^ cells) in RPMI 1640 medium supplemented with 5% fetal calf serum, 2 mM L-glutamine, 50 μM 2-mercaptoethanol, 1 mM sodium pyruvate, 1.5 mg/ml of sodium bicarbonate, and 25 mM HEPES (complete medium). The cells were allowed to adhere overnight at 37°C in a humidified 5% CO_2_ incubator. Non-adherent cells were then removed by washing several times with complete medium.

### Immunoassay and Western analysis

Macrophages from C57BL/6 WT mice were pre-incubated with medium only, rapamycin (3 h) or with selective inhibitors (1 h) followed by stimulation with *F. tularensis* LVS [multiplicity of infection (MOI)=20] for the indicated time periods. Macrophages derived from KO mice were stimulated with *F. tularensis* LVS for the indicated times. Cells were then washed with PBS and lysed as previously described [43,44]. Protein concentrations in whole cell extracts were assessed using the Micro BCA^TM^ Protein Assay Kit (Pierce, Rockford, IL). Equivalent amounts of protein from cell lysates were separated by SDS-PAGE on a 7.5 or 10% Tris–HCl gel (Bio-Rad Laboratories, Hercules, CA). Protein was electrotransferred to immobilon-P transfer membranes (Millipore, Bedford, MA) and Western analysis was carried out with specific antibodies against the total and/or phosphorylated forms of extracellular signal-regulated kinase (ERK) (Thr^202^/Tyr^204^), p38 mitogen-activated protein kinase (Thr^180^/Tyr^182^), Akt (Ser^473^), PI3K p85 (Tyr^458^/p^55^), p70S6K (Thr^389^) and (Thr^421^/Ser^424^), 4E-BP1 (Ser^65^), eI-F4E (Ser^209^), S6 (Ser^235/236^) and (Ser^240/244^) and rictor (Cell Signaling Technology, Inc. Danvers, MA). Detection of antibodies was carried out using horseradish peroxidase-linked rabbit or mouse anti-IgG antibody, followed by ECL Western blot detection reagents (GE Healthcare UK, Buckinghamshire, England). Densitometer scans of blots were done using the AlphaImager 2000 documentation and analysis system (Alpha Innotech, San Leandro, CA). In some experiments, cells were pre-incubated with the selective p38 MAPK inhibitor SB203580 (10 µM), the MEK1/2/ERK1/2 inhibitor UO126 (10 µM), the Akt inhibitor VIII (isoenzyme selective Akt1/2; 500 nM), the Raf1 inhibitor InSolution™ Raf1 Kinase Inhibitor I (1 µM), or the PLC inhibitor U73122 (3 µM) (EMD Milipore; Rockland, MA); or the Ca^2+^ chelator BAPTA-AM™ (10 µM), the PI3K inhibitor wortmannin (100 nM), or the mTOR inhibitor rapamycin (50 µg/ml) (Sigma-Aldrich; St. Louis, MO). The concentration of the inhibitors used was determined to be optimal in preliminary studies that tested different concentrations of each inhibitor. The effect of the inhibitors on macrophage viability was assessed by trypan blue exclusion, and on bacterial viability by microbiologic analysis as previously described [41]. The concentration of each inhibitor used in our study had no affect on macrophage or bacterial viablility. All experiments were repeated 3 to 5 times unless otherwise stated.

### Transfection assay

Murine macrophage-like RAW 264.7 cells were cultured in 96- or 24-well plates in complete medium until 50-70% confluency was reached. Cells cultured in 96-well plates were transfected with nonspecific siRNA or with siRNA (100 nM) targeted to the Akt1/2 or PLCγ1 genes (Santa Cruz, Santa Cruz, CA) using Lipofectamine™ RNAiMAX (0.3 µl), whereas cells cultured in 24-well plates were transfected with non-specific siRNA or siRNA targeted to the mTOR gene (Santa Cruz, Santa Cuz, CA) using Lipofectamine™ RNAiMax (1.5 µl) transfection reagent (Life Technologies, Grand Island, NY), according to the manufacturer’s instructions. Transfected cells were incubated at 37°C for 5 days and rested for 4 h prior to the addition of *F. tularensis* LVS. Cells were then washed, lysed and assessed by Western analysis.

### Immunoprecipitation assay

Macrophages from C57BL/6 WT mice were cultured in 60-mm cell culture plates overnight. Cells were pretreated with rapamycin (50 µg/ml; 3 h) or media only, followed by infection with *F. tularensis* LVS (MOI=20). Cells were washed twice with PBS and lysed with 500 µl of lysis buffer. Lysates were collected and assessed for protein content. Equal amounts of protein were transferred to microfuge tubes and 5 µl of anti-rictor antibody (0.1 mg/0.4 ml; Bethyl Labs, Montgomery, TX) or 5 µl of IgG control antibody (0.1 mg/ml; Santa Cruz) were added to the correspondent samples. Samples were rotated overnight at 4°C. Afterwards, 50 µl of protein A sepharose CL-4B (GE Heathcare UK, Buckinghamshire, England), prepared in a 50% slurry, was added to each sample and incubated for 2 h. Samples were then washed 3 times in lysis buffer and 45 µl of Laemmli buffer with 5% mercaptoethanol was added to each sample and assessed by Western analysis.

### 7-Methyl GTP Pull-down assay

Macrophages from C57BL/6 WT mice were cultured in 60-mm cell culture plates overnight. Cells were pretreated with rapamycin (50 µg/ml; 3 h) or media only, followed by infection with *F. tularensis* LVS (MOI=20). Cells were washed twice with PBS and lysed with 750 µl of TritonX buffer. Lysates were collected and assessed for protein content. Equal amounts of protein were transferred to microfuge tubes and 30 µl of 7-Methyl GTP sepharose (GE Heathcare UK, Buckinghamshire, England) was added to each sample. The samples were rotated for 2 h at 4°C. Samples were then washed 3 times in TritonX buffer, and 45 µl of Laemmli buffer with 5% mercaptoethanol was added to each sample and assessed by Western analysis.

### Bacterial invasion

Thioglycollate-induced peritoneal macrophages from WT mice were harvested, washed and cultured in complete medium at 5 x 10^5^ cells/well overnight in 24-well plates. Cells derived from WT mice were incubated or not with rapamycin (50 µg/ml; 3 h), U73122 (3 µM; 1 h), UO126 (10 µM; 1 h), SB203580 (10 µM; 1 h), Akt VIII (500 nM; 1 h), wortmannin (100 nM; 1 h), BAPTA (10 µM; 1 h) or cytochalasin D (2.5 µg/ml; 1 h) prior to incubation with bacteria. The indicated concentration of each inhibitor was shown to be optimal in preliminary studies. RAW cells transfected with control siRNA or with siRNA directed to the mTOR gene were cultured in complete medium in 24-well plates (see above). Freshly harvested *F. tularensis* LVS (MOI=20) were added to WT derived cells and to a portion of the transfected RAW cells for 90 min to assess bacterial invasion. Following incubation, cells were washed (5x) with PBS^+^ at room temperature and complete media containing gentamycin (50 µg/ml) was then added to the cultures for no more than 45 min to kill extracellular bacteria. Afterwards, cells were washed as described above and lysed for 5 min with ice-cold distilled water (150 µl). Lysates were serially diluted, plated on Mueller-Hinton II agar plates, and plates were incubated at 37°C in a 5% CO_2_ atmosphere [45]. Colonies were counted after 72-96 h. Non-transfected RAW cells treated or not with rapamycin were incubated or not with bacteria and used as controls. Each condition was set up in triplicate, and the experiment was repeated a minimum of three times. None of the inhibitors had an effect on macrophage viability as determined by trypan blue exclusion. In addition, preliminary studies demonstrated that the inhibitors had no effect on bacterial viability as determined by microbiologic analysis of bacterial suspensions incubated with or without each inhibitor. The number of internalized bacteria was expressed as a percent of the control infected cells [23]. Additionally, transfected RAW cells were washed following incubation with bacteria, as described above, and then the cells were lysed and lysates were assessed for the phosphorylation of mTOR downstream effectors by Western analysis.

### Fluorescence confocal microscopy

Cells were plated in 6-well plates with coverslips in each well and either left untreated or treated with rapamycin or wortmannin, as described above. One set of wells (control, +rapamycin or +wortmannin) was supplemented with buffer alone, while the other received *F. tularensis* (MOI= 20), and then all sets were incubated for 90 min. Cells were then quickly washed with ice-cold phosphate-buffered saline (PBS) and fixed with 3% paraformaldehyde in PBS for 10 min at room temperature followed by permeabilization with 0.1% Triton X-100 in PBS for 7 min at room temperature. The coverslips were then incubated for 45 min at room temperature with Phalloidin conjugated to Alexa-Fluor 594 (Molecular Probes, Eugene, OR) to stain actin and mouse monoclonal *F. tularensis* anti-lipopolysaccharide (LPS) antibody (FB11; 1:2000 dilution; Advanced Immunochemical Inc., Long Beach, CA) to visualize *F. tularensis* bacteria. Cells were washed with 0.2% Tween 20 in PBS and incubated for 30 min at room temperature with goat anti-mouse Alexa-Flour 488 secondary antibody (Molecular Probes, Eugene, OR). Cells were washed with 0.2% Tween 20 in PBS, nuclei were labeled with Hoescht stain and coverslips were mounted in 9:1 glycerol/PBS with 0.1% *p*-phenylenediamine (Sigma Aldrich, St. Louis, MO) on glass slides. Z-stack confocal images were acquired using a Nikon Eclipse TE 2000-U and Velocity 3D Image Analysis software (Perkin Elmer, Waltham, MA).

### Statistics

Statistical significance between infected control cells and infected cells pretreated with inhibitors was evaluated by Student’s two-tailed *t* test using the InStat program (Graphpad Software, San Diego, CA).

## Results

### mTOR signaling is involved in *F. tularensis* LVS invasion of primary macrophages.

**Figure 1:**
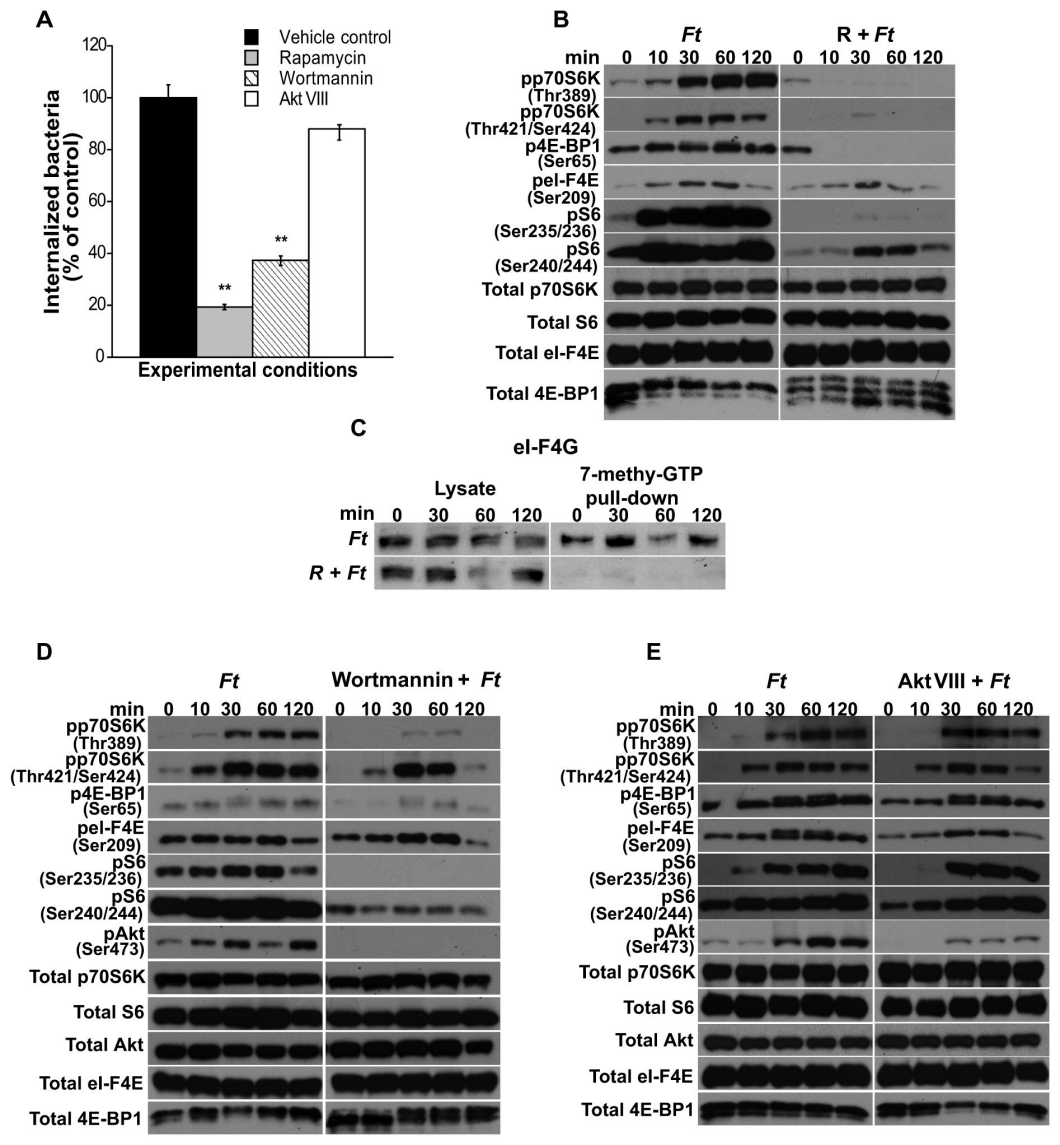
Effect of mTOR, PI3K and Akt inhibitors on the internalization of *F. tularensis* LVS. (A) Peritoneal macrophages derived from WT mice were pretreated or not with rapamycin (50 µg/ml; 3 h), wortmannin (100 nM; 1 h) or Akt VIII (500 nM; 1 h) and infected with freshly harvested *F. tularensis* LVS (MOI=20) for 90 min to assess bacterial invasion. Values are the mean ± SEM of 5 independent experiments, each done in triplicate; **p < 0.001; *p < 0.05 compared with infected control cells treated with DMSO. Peritoneal macrophages derived from WT mice were pretreated or not with the inhibitors as described above, exposed to *F. tularensis* LVS for 0-120 min and then lysed. (B, D, E) Total p70S6K, S6, 4E-BP1, eI-F4E and Akt, and phosphorylated p70S6K (Thr^389^ and Thr^421^/Ser^424^), 4E-BP1 (Ser^65^), S6 (Ser^235/236^ and Ser^240/244^), eI-F4E (Ser^209^) and Akt (Ser^473^) were assessed by Western analysis. Samples analyzed contained an equal amount of protein. Unstimulated control cells (time 0) were incubated with the respective inhibitors for the correspondent pre-incubation period. Prior to the addition of bacteria, cells were not washed including unstimulated controls. Unstimulated cells served as negative controls. (C) Peritoneal macrophages were pretreated with rapamycin (50 µg/ml) and exposed to *F. tularensis* LVS for 0-120 min. An equal amount of protein from each lysate was pulled-down using 7-methyl GTP sepharose beads. Pull-down products were assessed for eI-F4G by Western analysis. All gels are representative of three to five independent experiments.

To assess the involvement of mTOR signaling in the invasion of host cells by *F. tularensis* LVS, freshly isolated peritoneal macrophages were pretreated or not with the mTOR inhibitor rapamycin or with media containing DMSO (control). Cells were then co-cultured for 90 min with freshly harvested *F. tularensis* LVS and invasion was assessed. Rapamycin treatment of macrophages significantly reduced *F. tularensis* invasion to 19% compared to *F. tularensis* infected, untreated control cells (Figure 1A). Since mTOR is downstream of the PI3K/Akt pathway, we likewise assessed if signaling via PI3K was required for *F. tularensis* LVS invasion. Pretreatment of cells with the PI3K inhibitor wortmannin significantly reduced bacterial entry to 37% (Figure 1A), whereas inhibition of the PI3K downstream target Akt with the inhibitor Akt VIII (inhibitor of Akt1/2) did not significantly reduce the number of bacteria invading macrophages compared to infected, untreated controls (Figure 1A). No statistical difference was observed in bacterial invasion of host cells following treatment of cells with wortmannin or rapamycin. These results indicate that PI3K and mTOR signaling play important roles in *F. tularensis* LVS invasion of primary murine macrophages. Recent studies have demonstrated differences between *F. tularensis* LVS grown in Brain Heart Infusion broth (BHI) and that grown in Mueller-Hinton broth (MHB) [46,47]. Since the bacteria used in our studies were grown in MHB, we sought to verify that infection of cells with *F. tularensis* grown in BHI compared to those grown in MHB resulted in a similar phosphorylation pattern of mTOR downstream effector molecules, and a similar ability to invade macrophages. Therefore, macrophages were incubated with freshly harvested bacteria grown in BHI [46] or in MHB [41,42] for 90 min and invasion was assessed. No significant differences were seen in the number of bacteria internalized by macrophages using *F. tularensis* grown in BHI or that grown in MHB (Figure S1A). 

To delineate the phosphorylation events of the downstream mTOR signaling cascade that take place as a consequence of *F. tularensis* infection, peritoneal macrophages were pre-treated or not with rapamycin and then exposed to *Francisella* for various times. The phosphorylation of mTORC1 downstream effector proteins was then assessed as previously described [21,22,48,49]. Phosphorylation of p70S6K^Thr389^ induced by *F. tularensis* LVS infection was seen at 30 min, and was increased at 60 and 120 min, whereas p70S6K^Thr421/Ser424^ phosphorylation was detected at 10 min and peaked at 30 min (Figure 1B). However, when cell cultures were treated with rapamycin prior to the addition of bacteria, phosphorylation of p70S6K was suppressed (Figure 1B). Phosphorylation of S6 ribosomal protein, a downstream target of p70S6K, was also observed in cells infected with *Francisella* (Figure 1B); however, treatment of cells with rapamycin prior to the addition of bacteria abrogated phosphorylation of S6^Ser235/236^, whereas the level of phosphorylated S6^Ser240/244^ (pS6^Ser240/244^) detected was notably less than that observed in non-rapamycin treated, infected macrophages (Figure 1B). Similarly, phosphorylation of 4E-BP1, which involves the other arm of the mTORC1 downstream signaling cascade [29,30], was also suppressed in rapamycin-treated cells (Figure 1B). Following exposure of cell cultures not treated with rapamycin to bacteria, a peak in the level of 4E-BP1 phosphorylation was seen at 60 min, as evidenced by an upward shift in electrophoretic mobility (Figure 1B), consistent with previous studies establishishing that phosphorylation of specific sites on 4E-BP1 retards its electrophoretic mobility [50,51]. 4E-BP1 positively or negatively regulates the function of the 4F-translation initiation complex (eI-F4E) by reversibly associating with it. Specifically, hypo- or dephosphorylated 4E-BP1 avidly binds eI-F4E, thereby inhibiting its phosphorylation and translational activity, whereas when 4E-BP1 is phosphorylated, this interaction is disrupted, leading to eI-F4E activation [50,52]. Western analysis of phosphorylated eI-F4E (peI-F4E) in macrophages treated with rapamycin and infected with *F. tularensis* LVS revealed less phosphorylation at 60 and 120 min, compared to that seen with lysates of cells exposed to bacteria only (Figure 1B). Importantly, the resulting phosphorylation of the mTORC1 downstream proteins was not due to differences in total protein, except in the case of 4E-BP1 where rapamycin affects total 4E-BP1 (Figure 1B). It is noteworthy that phosphorylation of these downstream proteins was also blocked when rapamycin was removed from the cultures by washing prior to the addition of bacteria (not shown). Finally, the phosphorylation patterns of mTOR downstream effectors induced by *F. tularensis* LVS grown in BHI were similar to that seen with *F. tularensis* LVS grown in MHB (Figure S1B). 

To determine if the downregulatory effect on eI-F4E phosphorylation by rapamycin resulted in the dampening of the translation initiation machinery, we next carried out a 7-methyl GTP pulldown assay and assessed binding to eI-F4G by Western analysis. No eI-F4G was detected in lysates from cultures treated with rapamycin, suggesting that the absence of mTOR signaling with *F. tularensis* LVS infection does not allow the formation of the eI-F4F in spite of the low level of peI-F4E observed in the presence of rapamycin (Figure 1C).

To confirm that phosphorylation of mTOR downstream signaling molecules is critical for the internalization of *Francisella* into primary macrophages, RAW cells were transfected with siRNA control or siRNA to mTOR and exposed to *Francisella* for 90 min for the assessment of invasion and phosphorylation of mTOR downstream effectors. Transfection of cells with siRNA to mTOR resulted in a significant decrease (77%) in the number of invading bacteria, compared to that seen with siRNA control transfected RAW cells (Figure S2A), which was similar to that seen in primary macrophages (Figure 1A) and RAW (not shown) cells treated with rapamycin prior to the addition of bacteria. Moreover, the phosphorylation pattern of mTOR’s downstream effector proteins of cells transfected with siRNA to mTOR, but not with siRNA control, was similar to that observed in peritoneal macrophages treated with rapamycin prior to the addition of bacteria (Figure S2B). 

Next, we determined the role of PI3K signaling on the phosphorylation of mTOR downstream effectors in macrophages exposed to *Francisella* in the presence or absence of wortmannin. Our findings revealed that inhibition of PI3K signaling in *F. tularensis* LVS-infected macrophages by pretreatment with wortmannin abrogated phosphorylation of p70S6K at Thr^389^ and at Thr^421/Ser424^ (Figure 1D), as well as phosphorylation of 4E-BP1, but not phosphorylation of eI-F4E (Figure 1D). Furthermore, phosphorylation of S6 at Ser^235/236^ was also inhibited. A low level of pS6 at Ser^240/244^ was apparent at 30 and 60 min (Figure 1D), suggesting that a PI3K-independent pathway is also involved in the regulation of S6 phosphorylation at Ser^240/244^. Lastly, inhibition of PI3K also abrogated Akt^Ser473^ phosphorylation (Figure 1D). The above data shows, as previously reported [19,53-55], that inhibition of PI3K affected the phosphorylation of downstream targets of mTORC1; however, it further suggests that PI3K inhibition also affected mTORC2, since mTORC2 has been shown to regulate Akt^Ser473^ phosphorylation [31,56]. The observed phosphorylation of the respective proteins derived from cultures that were infected only or treated with wortmannin and then infected were not due to differences in total protein (Figure 1D). To further establish the direct involvement of Akt in the regulation of mTOR’s downstream molecules, we evaluated if chemical inhibition of Akt rendered similar results to those obtained with wortmannin. Downregulation of phosphorylated Akt^Ser473^ (pAkt^Ser473^) was achieved by use of the Akt inhibitor VIII in macrophages exposed to *Francisella*; however, essentially no change was seen in p70S6K phosphorylation at Thr^389^ or at Thr^421^/Ser^424^ or in the phosphorylation of 4E-BP1, eI-F4E or S6 at Ser^235/236^ or at Ser^240/244^ (Figure 1E). The resulting phosphorylation of the respective proteins derived from cultures infected only or treated with Akt VIII and then infected, was not due to differences in total protein (Figure 1E). To demonstrate that these results were indeed due to the specific inhibition of Akt, we next transfected RAW cells with siRNA to Akt and cultured them or not with *F. tularensis* (Figure S3). While complete abrogation of Akt^Ser473^ phosphorylation was seen (Figure S3), phosphorylation of p70S6K^Thr389^ was not suppressed as seen with wortmannin (Figure 1D). These studies suggest the participation of a PI3K-dependent, Akt-independent regulation of the downstream targets of mTORC1 following *Francisella* infection of primary macrophages. Taken together, the phosphorylation data of the mTORC1 downstream effector molecules in infected macrophages (Figures 1B, 1D, 1E and Figure S2B) and that of *Francisella* invasion in the presence or absence of inhibitors (Figures 1A and Figure S2A) indicate that mTORC1 downstream effector molecules are central for the infection of peritoneal macrophages by *F. tularensis* LVS. 

**Figure 2:**
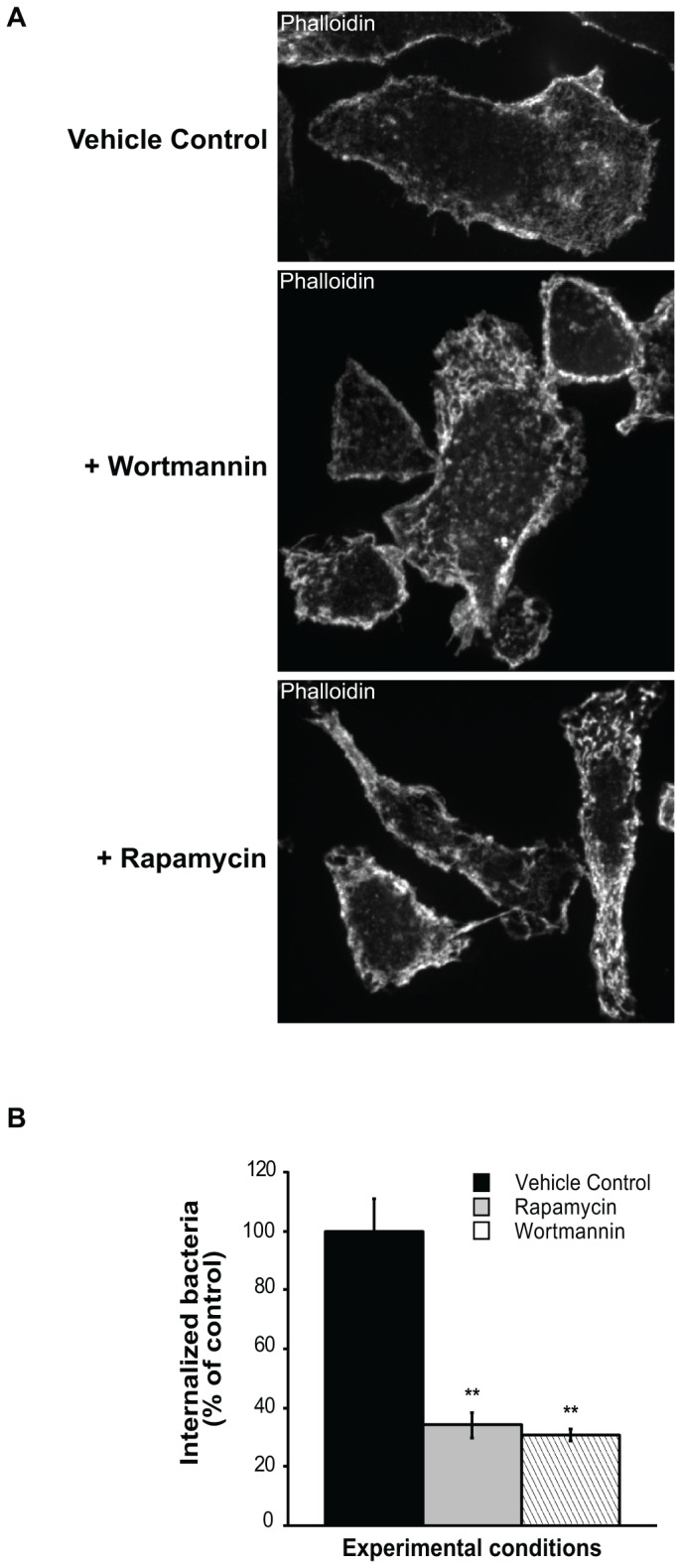
Effects of wortmannin and rapamycin on actin cytoskeleton and F. tularensis entry into cells. (A) Peritoneal macrophages were treated with vehicle control, wortmannin (1 h) or rapamycin (3 h) and stained with fluorescent β-phalloidin to detect F-actin. Wortmannin and rapamycin significantly alter the architecture of the actin cytoskeleton and induce numerous thick, short filaments and small patches distributed throughout the cell. Representative cells are shown. (B) Isolated macrophages were treated with vehicle control, wortmannin (for 1 h) or rapamycin (for 3 h), and then *F. tularensis* was added for 90 min. Cells were processed by immunofluorescence with an *F. tularensis* anti-LPS antibody to detect internalized bacteria. One hundred (100) cells for each treatment were counted in randomly selected fields. The number of bacteris in treated cells is expressed as percent normalized to the control (100%). The *p* values of rapamycin- and wortmannin-treated cells are <0.001 relative to control.

It has been reported that infection of brain endothelial cells by *Cronobacter sakazakii* disassembled actin fibers, but that cytochalasin D and PI3K inhibitors effectively blocked the bacterial effect on actin and cell invasion [57]. Furthermore, inhibition of mTOR signaling using rapamycin or stable inhibition of raptor (mTORC1) and rictor (mTORC2), decreased actin cytoskeleton remodeling [58]. Thus, we next assessed by fluorescence microscopy the effect of rapamycin and wortmannin, on the actin cytoskeleton. Our findings revealed that wortmannin and rapamycin significantly alter the architecture of the actin cytoskeleton by inducing numerous thick, short filaments and small patches distributed throughout the cell (Figure 2A). Moreover, rapamycin-treated and wortmannin-treated cells contained significantly fewer *F. tularensis* than control cells (Figure 2B). 

PLC and Ca^2+^ signaling play a role in the invasion of macrophages by *F. tularensis* LVS and in the regulation of downstream effectors of mTORC1 and mTORC2. 

**Figure 3:**
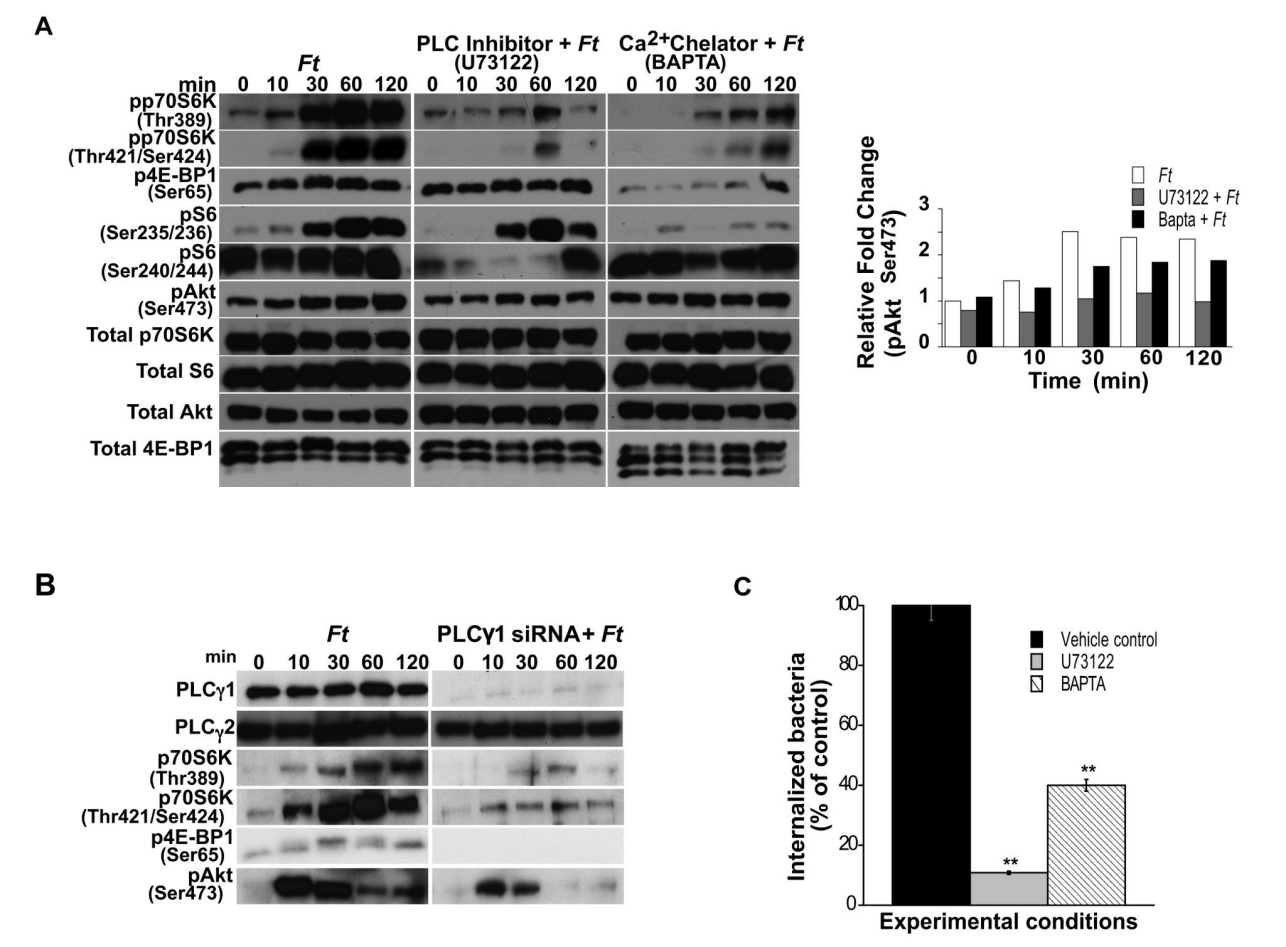
Effect of PLCγ1/Ca^2+^ signaling on mTOR downstream targets and *F. tularensis* host cell internalization. Peritoneal macrophages derived from WT mice were pretreated or not with U73122 (3 µM; 1 h) or BAPTA (10 µM; 1 h), infected with *F. tularensis* LVS (MOI=20) for 0-120 min and lysed. (A) Total p70S6K, S6, 4E-BP1 and Akt, and phosphorylated p70S6K (Thr^389^ and Thr^421^/Ser^424^), 4E-BP1 (Ser^65^), S6 (Ser^235/236^ and Ser^240/244^) and Akt (Ser^473^) were assessed by Western analysis. Samples analyzed contained equal amount of protein. (A) The band densities determined by densitometry for pAkt^Ser473^ were normalized to the total protein levels in a given lysate. Unstimulated control cells (time 0) were incubated with the respective inhibitors for the correspondent pre-incubation period. Prior to the addition of bacteria, cells were not washed including unstimulated controls. Unstimulated cells served as negative controls. (B) RAW cells were transfected with PLCγ1 siRNA (100 nM), and after 5 days, the cells were washed, rested and infected with *F. tularensis* LVS (MOI=20) for 0-120 min. Total PLCγ1 and PLCγ2, and phosphorylated p70S6K (Thr^389^ and Thr^421/Ser424^), 4E-BP1 (Ser^65^) and Akt (Ser^473^) were assessed by Western analysis. Samples analyzed contained equal amount of protein. Unstimulated cells served as negative controls. All gels are representative of three to five independent experiments. (C) Peritoneal macrophages derived from WT mice were pre-treated or not with U73122 or BAPTA, as described above, and exposed to freshly harvested *F. tularensis* LVS (MOI=20) for 90 min to assess bacterial invasion. Values are the mean ± SEM of 5 independent experiments, each done in triplicate; **p < 0.001; *p < 0.05 compared with infected control cells treated with DMSO.

Markova et al. [17] reported a novel Akt-independent signaling pathway regulating p70S6K activation via phospholipase Cγ1 (PLCγ1), Ca^2+^ signaling and protein kinase C in leukemic cells. Since our results support the notion of an Akt-independent phosphorylation of the mTORC1 cascade in peritoneal macrophages infected with *Francisella*, we questioned if PLC and Ca^2+^ signaling could play a role in the phosphorylation of the mTORC1 downstream effectors in our system. Cells were exposed to *Francisella* for various times after pre-incubation or not with the PLC inhibitor U73122 [59] or with the intracellular Ca^2+^ chelator 1,2-bis (o-aminophenoxy)ethane-N,N,N’N’-tetraacetic acid, sodium (BAPTA) (Calbiochem Biosciences Inc. Gibbstown, NJ), and then lysed and subjected to Western analysis. Treatment of cells with U73122 or BAPTA prior to their incubation with *F. tularensis* LVS decreased the level of phosphorylated p70S6K (pp70S6K) at Thr^389^ and at Thr^421^/Ser^424^, as well as that of 4E-BP1 (p4E-BP1) (Figure 3A). Downregulation of S6^Ser240/244^ phosphorylation was more evident in the presence of U73122 than of BAPTA (Figure 3A), whereas S6^Ser235/236^ phosphorylation was affected more by Ca^2+^ than by PLC signal inhibition, suggesting a differential regulation of these S6 phosphorylation sites by PLC and Ca^2+^ signaling in peritoneal macrophages exposed to *Francisella*. The resulting phosphorylation of the respective proteins derived from cultures infected only or treated with the inhibitors and then infected, was not due to differences in total protein, except in the case of 4E-BP1 when BAPTA was used since it seems to affect total 4E-BP1 (Figure 3A). The relative fold changes in phosphorylated proteins observed by densitometry analysis were normalized against the total protein level of the appropriate target protein in a given lysate (Figure 3A). 

PLC can be subdivided into β, γ, δ and ε isotypes [60]. PLCγ signaling can be activated downstream from PI3K through interactions between their SH2 and/or PH domains with phosphatidyl-inositol-3,4,5-triphosphate, thus linking the PI3K and the PLCγ/Ca^2+^ signaling pathways [34,35,61]. Considering that PLCγ1 is ubiquitously expressed [60] and is involved in the internalization of bacteria, e.g., *E. coli* [15], we next used siRNA against PLCγ1 to assess if this isotype played a role in the phosphorylation of molecules of the mTOR cascade as shown with the specific chemical inhibitor. Indeed, siRNA inhibition of PLCγ1, but not PLCγ2 in RAW cells infected with *Francisella*, resulted in downregulation of pp70S6K and p4E-BP1 (Figure 3B), in agreement with the findings obtained with U73122. These findings support a role for PLCγ1 in the activation of mTOR downstream effector molecules. Unlike the findings of Markova et al. [17], inhibition of PLC or Ca^2+^ signaling slightly downregulated the level of pAkt^Ser473^ (Figure 3A), and inhibition of PLCγ1 by siRNA yielded similar results (Figure 3B). The resulting phosphorylation of Akt^Ser473^ derived from cultures that were infected only or treated with inhibitor and then infected was not due to differences in total Akt protein. The relative fold changes in pAkt^Ser473^, as determined by densitometry analysis, were normalized against the total protein levels (Figure 3A). These findings suggest that PLCγ1 and Ca^2+^ signaling participate in the regulation of the downstream targets of mTOR in an Akt-dependent and -independent manner in peritoneal macrophages infected with *F. tularensis* LVS. Moreover, PLC and Ca^2+^ signaling play important roles in the internalization of *F. tularensis* into primary macrophages since their specific inhibition significantly reduced bacterial invasion to 11% and 40%, respectively, compared to *Francisella* infected control cells (Figure 3C).

The p38 and ERK1/2 pathways play critical roles in the regulation of mTOR signaling and their inhibition decreases *F. tularensis* invasion of primary macrophages. 

**Figure 4:**
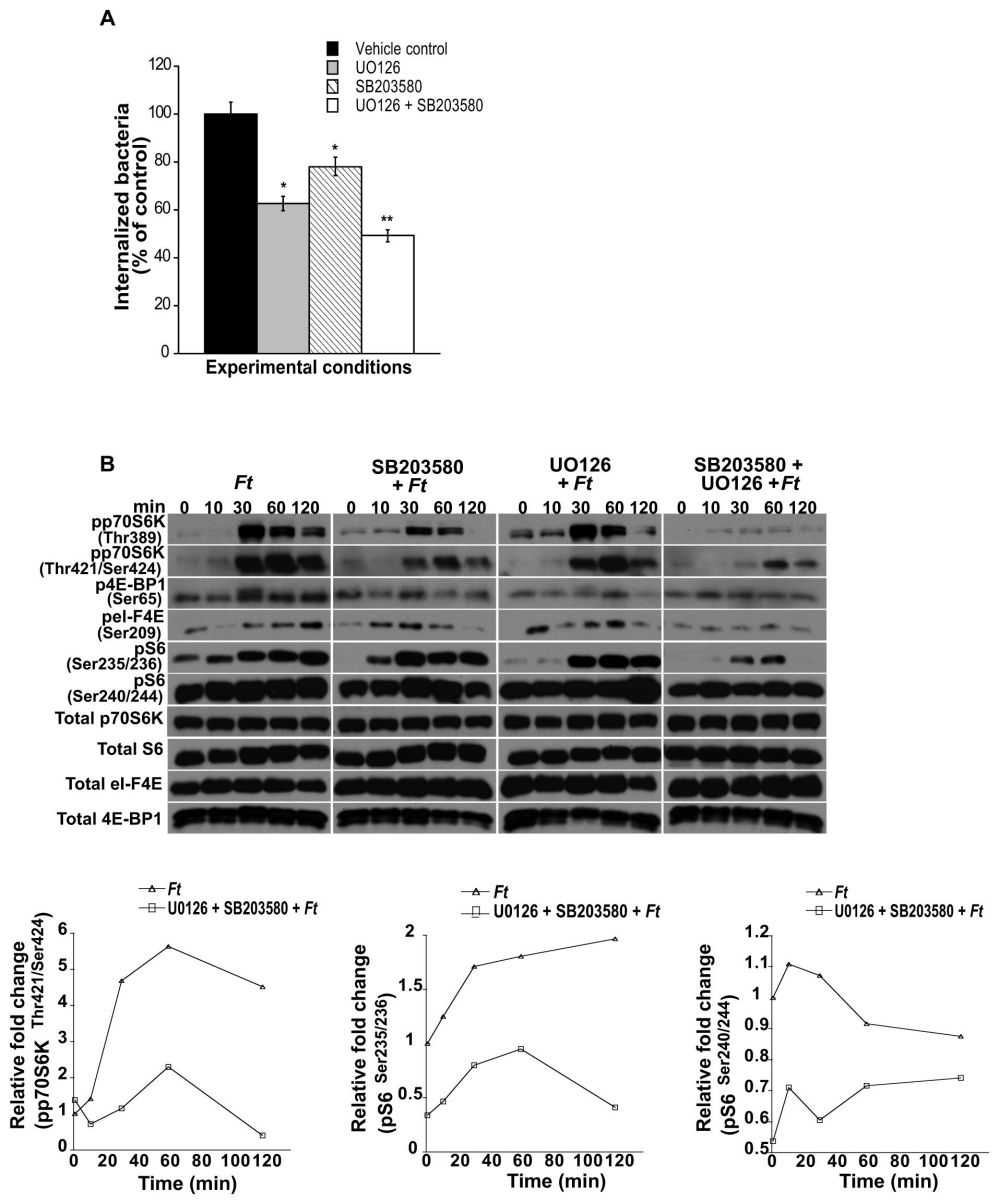
MEK/ERK and/or p38 inhibition affects *Francisella* cell invasion and phosphorylation of mTOR downstream effectors. (A) Peritoneal macrophages derived from WT mice were pretreated or not with UO126 (10 µM; 1 h), SB203580 (10 µM; 1 h) or with UO126 and SB203580 (1 h) and exposed to freshly harvested *F. tularensis* LVS (MOI=20) for 90 min to assess bacterial invasion. Values are the mean ± SEM of 5 independent experiments, each done in triplicate; **p < 0.001; *p < 0.05 compared with infected control cells treated with DMSO. Peritoneal macrophages derived from WT mice were pretreated or not with UO126, SB203580 or with a combination of both inhibitors as described above, exposed to *F. tularensis* LVS (MOI=20) for 0-120 min and then lysed. (B) Total p70S6K, S6, 4E-BP1 and eI-F4E, and phosphorylated p70S6K (Thr^389^ and Thr^421^/Ser^424^), 4E-BP1 (Ser^65^), S6 (Ser^235/236^ and Ser^240/244^) and eI-F4E (Ser^209^) were assessed by Western analysis. Samples analyzed contained equal amount of protein. Unstimulated control cells (time 0) were incubated with the respective inhibitors for the correspondent pre-incubation period. Prior to the addition of bacteria, cells were not washed including unstimulated controls. Unstimulated cells served as negative controls. (B) Band densities determined by densitometry were normalized to the total protein levels of the appropriate target protein in a given lysate. All gels are representative of three to five independent experiments.

Studies have demonstrated that ERK1/2 and p38 signaling is associated with actin regulation [8,62], and indeed their participation in host cell invasion by different pathogens has been reported [7,23,63]. Moreover, a role for ERK1/2 and p38 MAPK in the regulation of the mTOR pathway has been demonstrated through the activation of p70S6K, since the auto-inhibitory pseudosubstrate region in the C-terminal domain contains the consensus Ser/Thr-Pro sequence, and therefore, can be phosphorylated by members of the proline-directed protein kinases such as ERK1/2 and p38 [36,64]. Furthermore, the ERK1/2 and p38 pathways have been shown to be indirectly involved in the phosphorylation of the cap-binding protein eI-F4E. To first determine if ERK and p38 play a role in the host cell entry of *F. tularensis* LVS, peritoneal macrophages were pre-treated or not with inhibitors of MEK/ERK (UO126) or p38 (SB203580) signaling, exposed to bacteria, and then the number of invading *Francisella* assessed. Inhibition of MEK/ERK significantly decreased *F. tularensis* LVS host cell invasion to 63% compared to infected control cells (Figure 4A), lending support to the findings by Parsa et al. [7] that ERK plays a role in phagocytosis of *F*. *novicida*. In addition, our findings implicate for the first time p38 in the invasion process of *F. tularensis* LVS into primary macrophages, since inhibition of this MAPK significantly reduced bacterial internalization to 78% compared to *F. tularensis*-infected, untreated cells (Figure 4A). Moreover, the combined inhibition of MEK/ERK and p38 signaling resulted in a significant decrease (51%) of invading bacteria as compared to invasion by *F. tularensis* in untreated cells, suggesting a synergistic effect upon inhibition of these pathways (Figure 4A). Next, we determined if ERK and p38 signaling were involved in the phosphorylation events of the downstream targets of mTOR. Pretreatment of macrophages with UO126 or SB203580 prior to bacterial infection resulted in decreased levels of pp70S6K^Thr389^ compared to that seen with infected only cells, whereas pretreatment with both inhibitors completely abrogated p70S6K^Thr389^ phosphorylation (Figure 4B). Conversely, inhibition of either MEK/ERK or p38 resulted in a decrease in the levels of pp70S6K^Thr421/Ser424^, whereas inhibition of both MAPKs resulted in a marked downregulation in pp70S6K^Thr421/Ser424^ compared to *Francisella* infected control cells (Figure 4B). These findings indicate that while p38 and ERK are critically involved in the phosphorylation of p70S6K at Thr^389^, phosphorylation at Thr^421/Ser424^ is partially independent of these MAPKs. Furthermore, inhibition of ERK or p38 resulted in a decrease in the level of pS6^Ser235/236^ and dual inhibition of these MAPKs almost completely abolished it (Figure 4B). However, pretreatment with either UO126 or SB203580 had essentially no effect on the level of pS6^Ser240/244^, whereas a downregulatory effect was observed when both inhibitors were used (Figure 4B), suggesting that the p38 and the MEK/ERK pathways in peritoneal macrophages are primarily implicated in the regulation of S6 phosphorylation at Ser^235/236^, but not at Ser^240/244^ following *Francisella* infection. The phosphorylation pattern of the respective proteins derived from cultures infected only or treated with the inhibitors and then infected was not due to differences in total protein (Figure 4B). Densitometry analysis depicting relative fold changes in the indicated protein were normalized to the total protein levels of the respective target protein in the given cell lysate (Figure 4B). Lastly, inhibition of the p38 and MEK/ERK pathways similarly affected the other arm of mTORC1 downstream signaling because the concomitant use of p38 and MEK/ERK inhibitors downregulated further the level of p4E-BP1 and peI-F4E as compared to the effects exerted by each inhibitor alone (Figure 4B). Taken together, the effects exerted on the phosphorylation of mTOR downstream effectors in macrophages exposed to *Francisella* (Figure 4B), and on *F. tularensis* invasion (Figure 4A) in the presence or absence of pathway-specific inhibitors, SB203580 and/or UO126, suggest that the role played by p38 and MEK/ERK signaling in the invasion of primary macrophages by *Francisella* likely involves the mTOR downstream signaling cascade.

**Figure 5:**
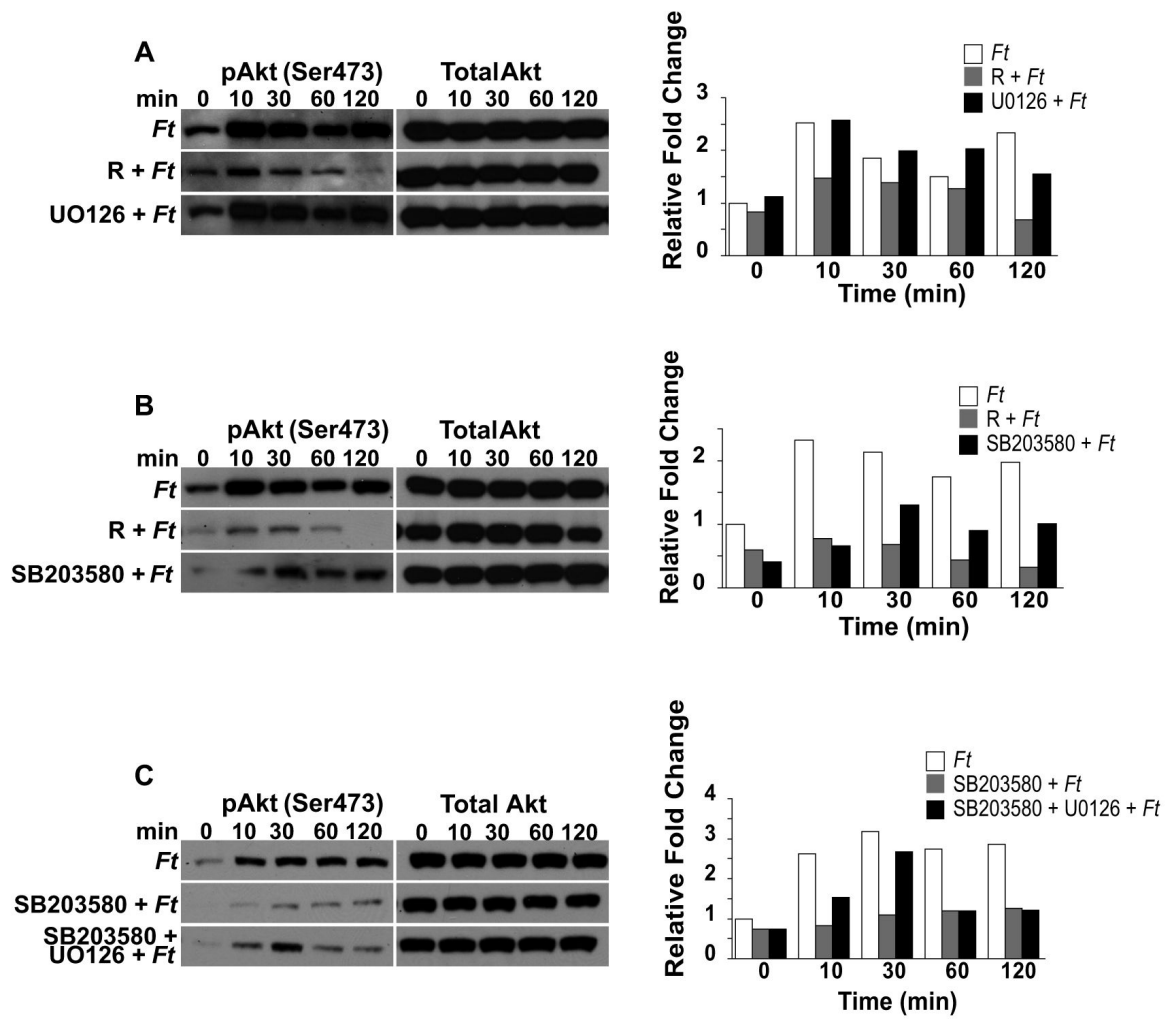
Inhibition of MEK and p38 differentially affect Akt (Ser^473^) phosphorylation. Peritoneal macrophages derived from WT mice were pretreated with rapamycin (50 µg/ml; 3 h) or UO126 (10 µM; 1 h); or with rapamycin or SB203580 (10 µM; 1 h); or with SB203580 or with UO126 and SB203580 (1 h), exposed to *F. tularensis* LVS (MOI=20) for 0-120 min and then lysed. (A, B, C) Total protein and phosphorylated Akt (Ser^473^) were analyzed by Western analysis. Samples analyzed contained equal amount of protein. Unstimulated control cells (time 0) were incubated with the respective inhibitors for the correspondent preincubation period. Prior to the addition of bacteria, cells were not washed including unstimulated controls. Unstimulated cells served as negative controls. (A, B, C) The band densities determined by densitometry were normalized to the total protein levels of the appropriate target protein in a given lysate. All gels are representative of three to five independent experiments.

**Figure 6:**
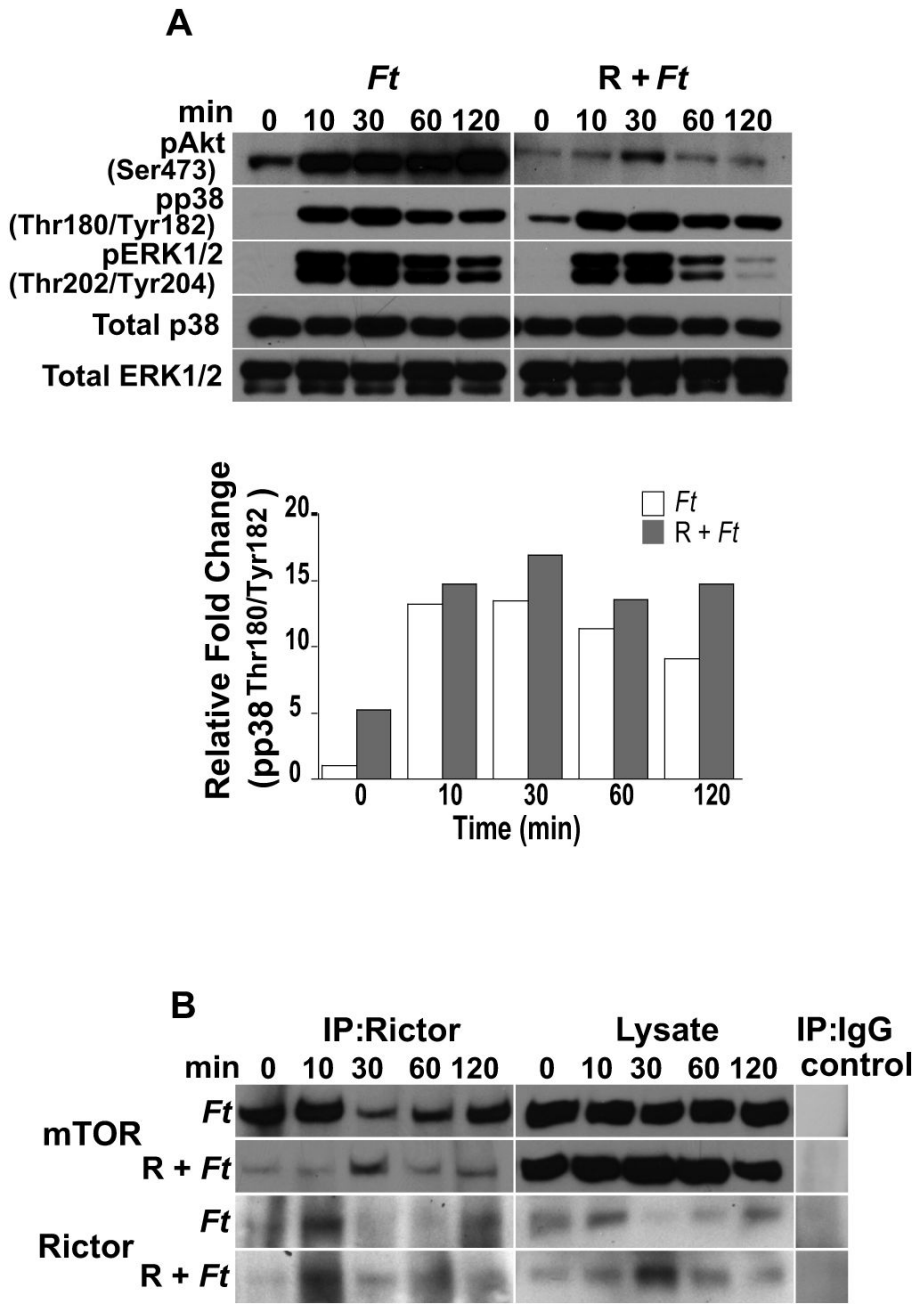
Rapamycin affects the phosphorylation of Akt (Ser^473^), ERK1/2 and p38, and the mTOR-rictor interaction. Peritoneal macrophages derived from WT mice were pretreated or not with rapamycin (50 µg/ml; 1 h), exposed to *F. tularensis* LVS for 0-120 min and then lysed. (A) Total p38 and ERK1/2, and phosphorylated Akt (Ser^473^), p38 (Thr^180^/Tyr^182^) and ERK1/2 (Thr^202^/Tyr^204^) were assessed by Western analysis. Samples analyzed contained an equal amount of protein. Unstimulated control cells (time 0) were incubated with rapamycin for the correspondent pre-incubation period. Prior to the addition of bacteria, cells were not washed including unstimulated controls. Unstimulated cells served as negative controls. The band densities determined by densitometry were normalized to the total protein levels of the appropriate target protein in a given lysate. (B) Peritoneal macrophages were pretreated or not with rapamycin and infected with *F. tularensis* LVS as described above. At the indicated times, cell lysates were prepared and immunoprecipitated with anti-rictor or IgG control antibody. Immunoprecipitates and lysates of mTOR and rictor from macrophages pretreated with rapamycin and infected with *F. tularensis* LVS or infected with bacteria alone were assessed by Western analysis. All gels are representative of three to five independent experiments.

Since Akt is an important component of the mTOR signaling cascade, we next determined if the p38 and/or MEK/ERK pathways are involved in the regulation of Akt^Ser473^ phosphorylation. Our findings revealed that while inhibition of MEK/ERK signaling had essentially no effect on Akt^Ser473^ phosphorylation (Figure 5A), inhibition of p38 resulted in a decrease in pAkt^Ser473^ levels. Although this effect was less pronounced than that observed with rapamycin (Figure 5B), the results suggest that p38 signaling is also involved in the regulation of Akt^Ser473^ phosphorylation. Unexpectedly, when SB203580 was used in combination with UO126, increased levels of pAkt^Ser473^ were observed compared to SB203580 alone (Figure 5C). The phosphorylation pattern of the respective proteins derived from cultures infected only or treated with the inhibitors and then infected was not due to differences in total protein (Figures 5A, 5B, 5C). Densitometry analysis depicting relative fold changes in the indicated protein were normalized to the total protein levels of the respective target protein in the given cell lysate (Figures 5A, 5B, 5C). Since our findings revealed the importance of p38 and ERK1/2 MAPKs in the modulation of mTOR downstream effector molecules, we next determined if mTOR signaling is involved in the phosphorylation of Akt^Ser473^, p38 and ERK in peritoneal macrophages exposed to *F. tularensis*. Downregulation in the level of pERK1/2 and pAkt^Ser473^ was observed in rapamycin treated cells compared to non-treated host cells that were exposed to *Francisella* for various periods of time (Figure 6A). However, an increase in the level of phosphorylated p38 (pp38) was observed in rapamycin-treated cells compared to that seen in infected only macrophages (Figure 6A). These results suggest that these molecules are downstream of mTOR or that a molecule(s) mediated by mTOR could act back on upstream signaling pathways. Furthermore, the observed downregulation of pERK1/2 and pAkt^Ser473^ (Figure 6A) was in line with a report demonstrating that mTORC2 is involved in the modulation of these molecules and that rapamycin downregulated their phosphorylation [56]. The phosphorylation pattern of the respective proteins was not due to differences in total protein (Figure 6A). Immunoprecipitation of rictor in rapamycin-treated, but not in non-treated cells, revealed reduced levels of mTOR (Figure 6B), suggesting, as previously reported, that rapamycin partially affects the mTOR-rictor interaction [31]. Lastly, our findings suggest that mTOR signaling is a negative regulator of p38 phosphorylation as indicated by densitometry analysis (Figure 6A). 

TLR2 signaling is necessary for the involvement of the mTOR cascade and MAPK in *Francisella* infection of peritoneal macrophages. 

**Figure 7:**
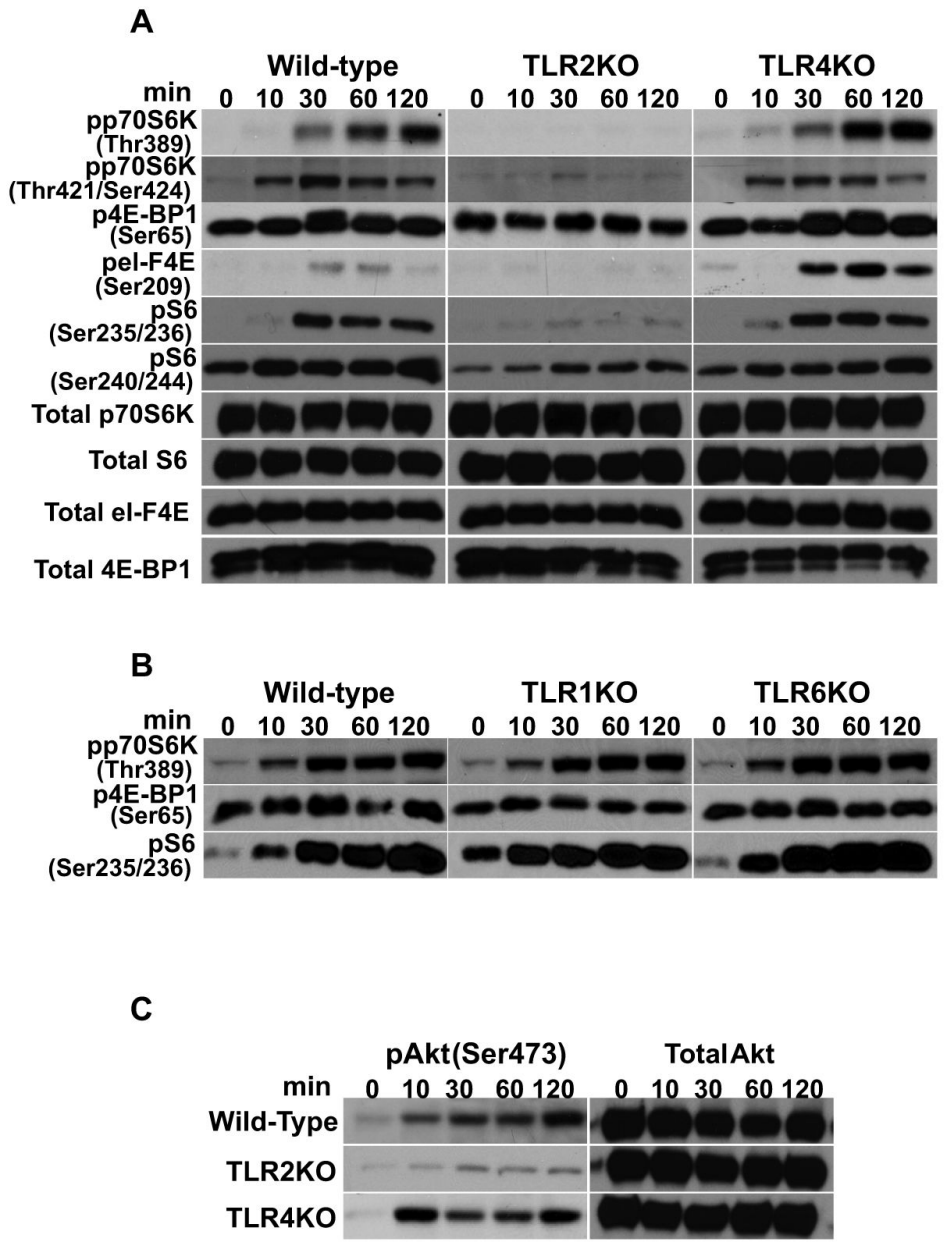
TLR2 is necessary for the phosphorylation of the mTOR downstream signaling cascade upon *Francisella* infection. Peritoneal macrophages derived from WT, and TLRKO mice were exposed to *F. tularensis* LVS (MOI=20) for 0-120 min and then lysed. Total p70S6K, S6, 4E-BP1 and eI-F4E, and phosphorylated (A) p70S6K (Thr^389^ and Thr^421^/Ser^424^), 4E-BP1 (Ser^65^), S6 (Ser^235/236^ and Ser^240/244^) and eI-F4E (Ser^209^) were assessed in WT, TLR2KO and TLR4KO derived macrophages by Western analysis; (B) Phosphorylated p70S6K (Thr^389^) 4E-BP1 (Ser^65^) and S6 (Ser^235/236^) in WT, TLR1KO and TLR6KO derived macrophages was assessed by Western analysis; (C) Total protein and phosphorylated Akt (Ser^473^) in WT, TLR2 and TLR4KO derived macrophages were assessed by Western analysis. Samples analyzed contained an equal amount of protein. (A) Phosphorylation of the respective proteins was not influenced by differences in total protein in TLRKO cells. Unstimulated cells served as negative controls. All gels are representative of three to five independent experiments.

TLRs are pattern recognition receptors that can distinguish molecules broadly shared among microbial pathogens. Recognition of these molecules via TLRs triggers the activation of downstream signaling cascades. Since it had been previously shown that *F. tularensis* LVS is a TLR2/6 and TLR2/1 agonist [42,65], we next determined the importance of TLR2, TLR1 and TLR6 in the phosphorylation events of mTORC1 downstream effectors. Exposure of TLR2KO macrophages to *Francisella* resulted in a lack of pp70S6K^Thr389^, pp70S6K^Thr421/Ser424^, p4E-BP1, peI-F4E, pS6^Ser235/236^ and a reduction in pS6^Ser240/244^ compared to that seen with WT and TLR4KO cells (Figure 7A). Assessment of the corresponding total protein levels in TLR2KO and WT macrophages revealed no differences, indicating that the lack or reduced phosphorylation was not due to a decrease in total protein (Figure 7A). Interestingly, *F. tularensis* infection of TLR4KO macrophages resulted in an increase in peI-F4E compared to that seen with WT cells, suggesting that an *F. tularensis*-induced TLR4 signal possibly limits phosphorylation of eI-F4E in WT macrophages (Figure 7A). Our findings further revealed that TLR1 and TLR6 signaling is not necessary for the phosphorylation events of the mTORC1 downstream signaling cascade, as macrophages derived from these KO mice exposed to bacteria showed levels of pp70S6K^Thr389^, p4E-BP1 and pS6^Ser235/236^ comparable to the levels detected in WT cells (Figure 7B). These findings indicate that TLR2 is the main pattern recognition receptor involved in the regulation of mTORC1 downstream effector molecules in primary macrophages exposed to *F. tularensis* LVS. Furthermore, while comparable levels of pAkt^Ser473^ were seen in WT and TLR4KO macrophages exposed to *Francisella*, the level of pAkt^Ser473^ in TLR2KO cells was downregulated (Figure 7C), suggesting that TLR2 signaling also plays a role in mTORC2 Akt^Ser473^ phosphorylation. Levels of pAkt^Ser473^ in TLR1KO and TLR6KO cells were comparable to those observed in WT and TLR4KO macrophages (not shown).

**Figure 8:**
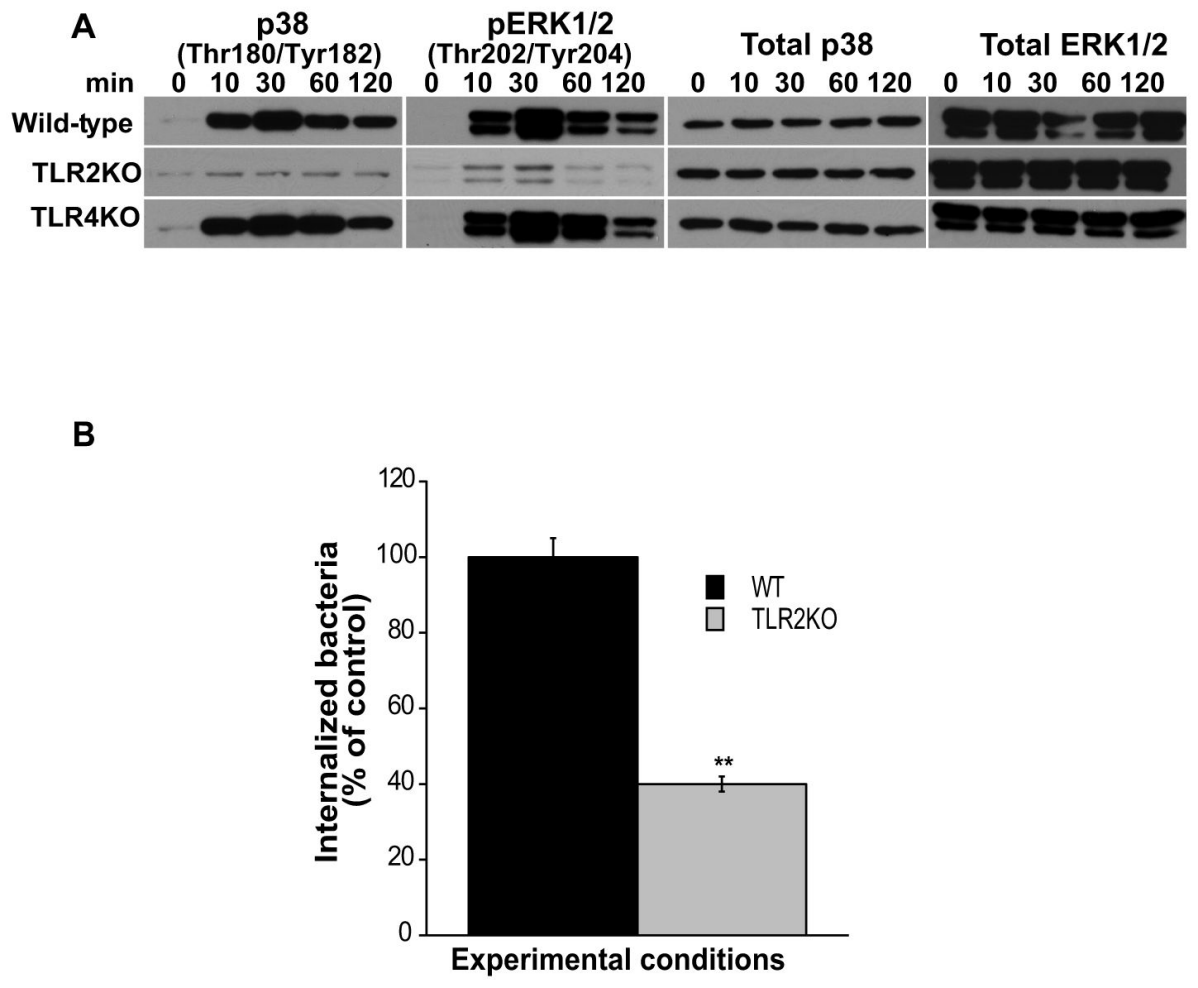
TLR2 signaling is important in *Francisella* invasion of primary cells. (A) Peritoneal macrophages derived from WT, TLR2KO and TLR4KO mice were exposed to *F. tularensis* LVS (MOI=20) for 0-120 min and then lysed. Total protein and phosphorylated p38 (Thr^180^/Tyr^182^) and ERK1/2 (Thr^202^/Tyr^204^) were assessed by Western analysis. Samples analyzed contained an equal amount of protein. Unstimulated cells served as negative controls. All gels are representative of three to five independent experiments. (B) Peritoneal macrophages derived from WT and TLR2KO mice were exposed to freshly harvested *F. tularensis* LVS (MOI=20) for 90 min to assess bacterial invasion. Values are the mean ± SEM of 5 independent experiments, each done in triplicate; **p < 0.001; *p < 0.05 compared with infected control cells treated with DMSO.

Next, we determined if the lack of TLR2 signaling that resulted in 4E-BP1 dephosphorylation and abrogation of p70S6K, S6 and eI-F4E phosphorylation (Figure 7A) was related to a lack of phosphorylation of p38 and/or the MEK/ERK pathway, since these play a critical role in the activation of the mTOR signaling cascade (Figure 4B). Exposure of TLR2KO macrophages to *F. tularensis* did not induce phosphorylation of p38 and ERK1/2 MAPK, whereas phosphorylation was seen in WT and TLR4KO macrophages at 10, 30 and 60 min following bacterial stimulation (Figure 8A). The lack of phosphorylation of these MAPK in TLR2KO macrophages was not due to a lack or decrease in total protein, as reflected by the comparable levels of total ERK and p38 proteins detected in WT, TLR2KO and TLR4KO macrophages (Figure 8A). These results indicate that in primary macrophages exposed to *F. tularensis* LVS, TLR2 signaling is necessary for the regulation of mTOR’s downstream effector molecules via the p38 and MEK/ERK pathways. Since downregulation in the phosphorylation of mTOR downstream effector targets was seen in TLR2KO cells, we predicted that reduced bacterial entry would be seen upon exposure of TLR2KO derived macrophages to *Francisella*. Indeed, the ability of the bacteria to invade TLR2KO compared to WT macrophages was significantly reduced to approximately 40% (Figure 8B). Taken together, the studies suggest that TLR2 signaling by *F. tularensis* LVS is necessary for the involvement of the mTOR downstream signaling cascade, important for *Francisella* invasion of primary macrophages. 

## Discussion

Intracellular pathogenic bacteria have developed strategies by which they manipulate host cell signaling pathways to facilitate their invasion, intracellular multiplication and survival. The participating downstream signaling events, directly or indirectly, exert a regulatory effect on the host cytoskeleton critical for the internalization of bacteria. In the present study, we have analyzed signaling molecular events in the context of the mTOR downstream signaling cascade in the internalization of *F. tularensis* LVS into primary macrophages.

Remodeling of the actin cytoskeleton is central to the invasion of host cells by bacteria, including that of *F. tularensis*, since treatment of peritoneal macrophages with the depolymerizing agent cytochalasin D prior to bacterial exposure essentially abolished *Francisella* host cell entry (not shown). Furthermore, inhibition of mTOR or of PI3K signaling by rapamycin or wortmannin, respectively, resulted in an alteration of the actin cytoskeleton architecture and inhibited the phosphorylation of mTOR downstream effector molecules. Moreover, in the presence of rapamycin or wortmannin *F. tularensis* invasion of primary macrophages was significantly reduced. Studies have shown that mTOR and downstream effector molecules are involved in actin cytoskeleton reorganization. For instance, inhibition of mTOR signaling using rapamycin or stable inhibition of raptor (mTORC1) and rictor (mTORC2), decreased actin cytoskeleton remodeling [58]. Moreover, Breven et al. [66] demonstrated that p70S6K, and activated mTOR were enriched at the actin arc of Swiss3T3 fibroblasts suggesting a role in actin cytoskeleton reorganization, and p70S6K was shown to be an important regulator of actin cytoskeleton by decreasing the rate and extent of actin depolymerization [11,67]. Furthermore, enhancement of phagocytosis by the endogenous lipid mediator Resolvin E1 involves the phosphorylation of the ribosomal protein S6 [25], and both p70S6K and 4E-BP1 were involved in the regulation of F-actin reorganization via the mTOR-raptor complex [68]. The mTOR downstream signaling cascade is also regulated by the PI3K pathway, located upstream of mTOR, and inhibition of PI3K by wortmannin abrogates phosphorylation of mTOR downstream effector molecules [9,53], a finding also observed in our studies. Furthermore, inhibition of PI3K significantly inhibited *C*. *sakazakii* invasion of brain endothelial cells and prevented actin rearrangement necessary for invasion [57].

While inhibition of rictor (mTORC2) decreased actin cytoskeleton remodeling [58], and Akt was shown to promote phagocytosis via the activation of p70S6K [69], in our studies, chemical inhibition of Akt with the specific inhibitor Akt VIII did not significantly decrease the number of internalized *F. tularensis* in primary macrophages, and not surprisingly, siRNA to Akt or chemical inhibition of Akt did not abrogate phosphorylation of mTOR downstream effector molecules, including that of p70S6K. These observations suggested an Akt-independent mechanism for the phosphorylation of mTOR downstream signaling molecules. These findings gain support from studies demonstrating that phosphorylation of mTOR downstream effectors was Akt-independent in leukemic cells [17], transformed B lymphocytes [70] and prostaglandin-F2α-treated bovine luteal cells [71]. However, to our knowledge, this is the first report showing that exposure of primary immune cells to F. tularensis LVS results in the phosphorylation of mTOR downstream effectors in an Akt-independent manner. 

Analysis of mRNA transcripts in rapamycin-treated cells demonstrated downregulation of several transcripts linked to phagocytosis [72]. It is known that treatment of cells with rapamycin limits the availability of eI-F4E via its sequestration into an inactive complex with the hypophosphorylated 4E-BPs [72]. While such an event can repress global translation rates, the most affected mRNAs containing TOP elements were always downregulated and involved transcripts implicated in phagocytosis [72]. Interestingly, Syk was one of those transcripts [72], and Syk has been found to play a role in the invasion of host cells by *Francisella* [7]. Moreover, macrophages treated with rapamycin, but not with wortmannin, and exposed to *F. tularensis* LVS, revealed an apparent downregulation of ERK1/2 phosphorylation. This MAPK is the downstream effector of Syk and was implicated by Parsa et al. [7] and supported by our findings to be involved in *F. tularensis* host cell infection. Indeed, the MEK/ERK pathway has been implicated in host cell entry by other pathogens like *C. pneumoniae* [23]. In addition to the involvement of the MEK/ERK pathway in the invasion process of *F. tularensis* into host cells shown in the present study and that of others [7], our findings revealed that p38 signaling also plays a role in *Francisella* invasion of peritoneal macrophages. The pathogen *Rickettsia rickettsii* has been shown to use p38 signaling for host cell entry [63]. Furthermore, it has been reported that activation of p38 can increase the stability of actin microfilaments in the presence of the actin depolymerizing agent cytochalasin D [62]. During the exposure of host cells to stress, p38 signaling may mediate an increased stability of the actin cytoskeleton, thus constituting an important event of the cell response to external stimuli [62]. Along these lines, *F. tularensis* LVS infection of macrophages induced the phosphorylation of p38, but in the presence of rapamycin, as shown in this study and by others [58,68], alteration of the actin cytoskeleton was observed and the level of pp38 was increased, suggesting that rapamycin contributes to the cell’s stress. Our findings further revealed that while inhibition of p38 and or MEK/ERK significantly decreased the number of *F. tularensis* invading macrophages, it also resulted in the downregulation of pp70S6K and pS6, and that the simultaneous inhibition of these signaling molecules, had an additive effect. The involvement of the MEK/ERK and p38 pathways in the regulation of the mTOR downstream effector molecules was not unexpected, since various studies favor a role for MAPK in the phosphorylation of the S/T-P (proline-directed serine/threonine) sites in the autoinhibitory C-terminal domain for the modulation of p70S6K [36,64]. Activation of p70S6K initially requires that the interaction between the p70S6K C-terminal domain containing an auto-inhibitory pseudosubstrate region and the N-terminal domain be relieved, since it is thought that the inactive conformation of p70S6K is in place when the acidic N-terminal domain interacts with the basic C-terminal region [64]. This allows for a hierarchical phosphorylation at multiple sites, such as at Thr^421^/Ser^424^ in the C-terminus, consequently loosening the conformation and permitting the phosphorylation of Thr^389^ [73]. 

Differential requirements for ERK1/2 and p38 on p70S6K phosphorylation have been observed contingent on the stimuli. For instance, UV-irradiation induced phosphorylation of p70S6K^Thr389^ was abrogated by ERK1/2 and p38 inhibitors, while that at Thr^421^/Ser^424^ was blocked by ERK1/2, but not p38 inhibition [74]. In addition, upon cell stimulation with amino acids, phosphorylation of p70S6K at Thr^421^/Ser^424^, but not at Thr^389^ was observed, and only the dual use of the inhibitors UO126 and SB203580 suppressed phosphorylation at these sites [75]. These reports, along with our findings using live bacteria, provide support that the nature of the stimulus is a relevant determinant for the differential participation of ERK1/2 and/or p38 in the regulation of p70S6K phosphorylation. Moreover, the involvement of MEK/ERK in the phosphorylation of the 40S ribosomal protein S6 at Ser^235/236^ seen in our studies is supported by the findings of Roux et al. [76], although these investigators did not show an involvement of p38 at this phosphorylation site, as reported in the present study and by other investigators [75]. Interestingly, p38, but not ERK1/2 signaling exert a regulatory effect on Akt^Ser473^ phosphorylation, yet inhibition of the MEK/ERK and p38 pathways in cells exposed to *Francisella* resulted in an increase in the level of pAkt^Ser473^, as compared with that seen by the inhibition of p38 only. While it has been previously reported that p38 regulates Akt^Ser473^ phosphorylation via MK2 (MAPKAPK-2), and that inhibition of p38 by SB203580 downregulates this process [77,78], how can we explain the increase in the level of pAkt^Ser473^ upon suppression of the MEK/ERK and p38 pathways? Menges et al. [79] showed that in cell arrest Raf via MEK/ERK inhibits Akt phosphorylation. Thus, in the presence of UO126, the ability of Raf to inhibit Akt phosphorylation would be lost. Indeed, inhibition of Raf in the presence of SB203580 resulted in increased levels of pAkt^Ser473^ (data not shown), hence supporting this notion. Given the reported involvement of p38 in the induction of G1/S and G2/M cell cycle checkpoints in response to stimuli [80-82], inhibition of p38 signaling could cause cell cycle arrest, and under these circumstances, Raf via MEK/ERK would have exerted a downregulatory effect on Akt^Ser473^ phosphorylation, as observed in our studies.

The involvement of phospholipases in phagocytic signaling has been well documented [83]. Phosphorylation of PLCγ1 was shown to be critical for the invasion of endothelial cells by *E. coli* [15]. The involvement of PLC, and specifically PLCγ1, in the regulation of the downstream targets of mTOR in primary cells in the context of a bacterial infection suggests that such modulation could account, at least partially, for the significant reduction in *Francisella* host cell entry when cells were pre-treated with the PLC inhibitor U73122. Since the cell permeable molecule BAPTA is enzymatically converted to the non-permeable, calcium chelator BAPTA inside the cell, only intracellular calcium is chelated [84]. Thus, our data suggests that PLC/PLCγ1 downstream signaling involved intracellular calcium, which also plays an important role in the invasion of *F. tularensis*. Furthermore, this inhibition was associated with a downregulated phosphorylation of mTORC1 downstream effectors as shown in other systems [17].

TLRs are highly conserved pattern recognition receptors and their recognition of pathogen associated molecular patterns induces the activation of host signal transduction pathways that result in an immune response that ultimately will aid in bacterial clearance [85]. However, in recent years, it has been reported that TLR signaling promotes phagocytic activity through the upregulation of genes involved in phagocytosis [86]. Moreover, antibodies to TLR4 inhibit internalization of *E. coli* by epithelial cells, and no bacteria could be recovered after pre-treatment with cytochalasin D or with antibodies against TLR4, suggesting that TLR4 participates in downstream signaling events that mediate bacterial internalization [87]. Furthermore, TLR2, but not TLR4, has been shown to be essential for efficient internalization of *Aspergillus fumigatus* conidia, since infection of macrophages deficient in TLR2 rendered reduced conidial cell entry [88]. Results of our studies show that TLR2 signaling is significantly implicated in the internalization of *F. tularensis* LVS into primary macrophages, and, in the context of our investigations, the involvement of downstream effector molecules of mTOR, as well as signaling molecules associated with their regulation via TLR2 signaling, are important for this process. 

Overall, and to the best of our knowledge, our findings reveal for the first time that the integrity of the actin cytoskeleton architecture and the phosphorylation of mTOR downstream effector molecules via mTOR and PI3K signaling, are central for the invasion of *F. tularensis* LVS into primary murine macrophages through TLR2 signaling. Moreover, the phosphorylation of mTOR downstream proteins via PLC/Ca^2+^ and MAPKs signaling was also shown to play a role in *Francisella* invasion of macrophages (Figure S4).

## Supporting Information

Figure S1 ***F. tularensis* LVS grown in BHI or in MHB show similar bacterial host cell entry.** (A) Peritoneal macrophages derived from WT mice were exposed to freshly harvested *F. tularensis* LVS (MOI=20) grown in Brain Hear Infusion broth (BHI) or in Muller-Hinton broth (MHB) for 90 min to assess bacterial invasion. (B) Peritoneal macrophages were exposed to *F. tularensis* LVS grown in BHI or MHB for 0-120 min and then lysed. Total p70S6K and phosphorylated p70S6K (Thr^389^ and Thr^421^/Ser^424^), 4E-BP1 (Ser^65^), S6 (Ser^235/236^ and Ser^240/244^) and eI-F4E (Ser^209^) were assessed by Western analysis. Samples analyzed contained an equal amount of protein. Unstimulated cells (time 0) served as negative controls. The gel is representative of three to five independent experiments.
(TIF)

Figure S2 **mTOR siRNA decreased internalization of *Francisella* and downregulates phosphorylation of mTOR downstream signaling cascade.** (A) RAW cells were transfected with control siRNA or with siRNA to mTOR (100 nM), and after 5 days, cells were washed, rested and infected with *F. tularensis* LVS for 90 min to assess bacterial invasion. Values are the mean ± SEM of 5 independent experiments, each done in triplicate; **p < 0.001; *p < 0.05 compared with infected control transfected with control siRNA. (B) RAW cells were transfected with control siRNA or with siRNA to mTOR (100 nM) or non-transfected, and after 5 days, cells were washed, rested, infected with *F. tularensis* LVS (MOI=20) for 0-90 min and then lysed. Total p70S6K and Akt, and phosphorylated p70S6K (Thr^389^ and Thr^421^/Ser^424^), 4E-BP1 (Ser^65^), S6 (Ser^235/236^ and Ser^240/244^), eI-F4E (Ser^209^) and Akt (Ser^473^) were assessed by Western analysis. Samples contained equal amount of protein. RAW cells transfected with control siRNA were used as negative controls. Gels are representative of three to five independent experiments.
(TIF)

Figure S3 **Phosphorylation of p70S6K in RAW cells transfected with siRNA to Akt1/2.** RAW cells were transfected or not with siRNA to Akt1/2 (100 nM), and after 5 days, cells were washed, rested, infected with *F. tularensis* LVS (MOI=20) for 0 and 120 min and lysed. Total and phosphorylated p70S6K (Thr^389^) and Akt (Ser^473^) was assessed by Western analysis. Samples analyzed contained equal amount of protein. Unstimulated cells served as negative controls. Gels are representative of three to five independent experiments.
(TIF)

Figure S4 **Proposed model of the signaling pathways involved in the phosphorylation of mTOR downstream effector molecules associated with *F. tularensis* invasion of primary macrophages via TLR2 signaling.**
(TIF)
