# Supplementary figures and images for: Role of mTOR Downstream Effector Signaling Molecules in Francisella Tularensis Internalization by Murine Macrophages
Source: PLoS One. 2013 Dec 3;8(12):e83226. doi: 10.1371/journal.pone.0083226 (PMC3849438; doi:10.1371/journal.pone.0083226)

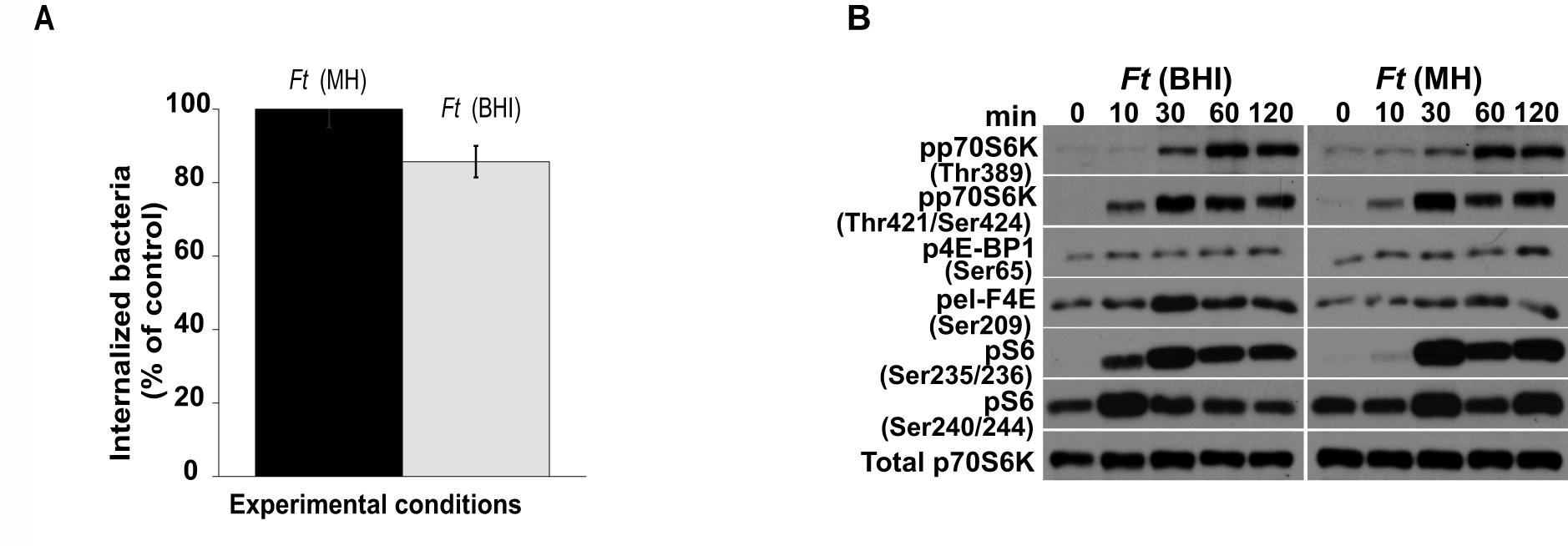

Supplement: Figure S1 — F. tularensis LVS grown in BHI or in MHB show similar bacterial host cell entry. (A) Peritoneal macrophages derived from WT mice were exposed to freshly harvested F. tularensis LVS (MOI=20) grown in Brain Hear Infusion broth (BHI) or in Muller-Hinton broth (MHB) for 90 min to assess bacterial invasion. (B) Peritoneal macrophages were exposed to F. tularensis LVS grown in BHI or MHB for 0-120 min and then lysed. Total p70S6K and phosphorylated p70S6K (Thr389 and Thr421/Ser424), 4E-BP1 (Ser65), S6 (Ser235/236 and Ser240/244) and eI-F4E (Ser209) were assessed by Western analysis. Samples analyzed contained an equal amount of protein. Unstimulated cells (time 0) served as negative controls. The gel is representative of three to five independent experiments. (TIF) [file pone.0083226.s001.tif]

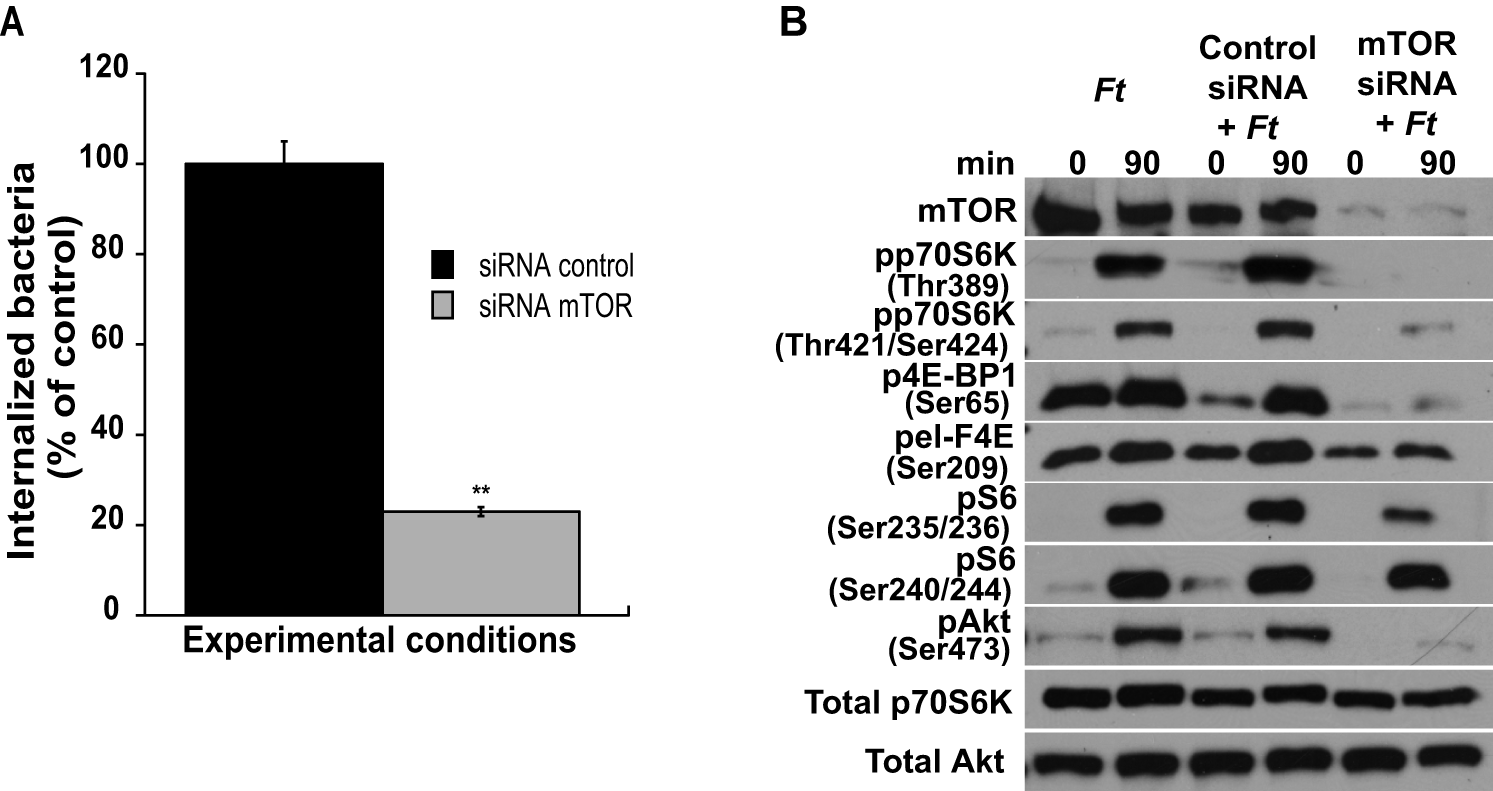

Supplement: Figure S2 — mTOR siRNA decreased internalization of Francisella and downregulates phosphorylation of mTOR downstream signaling cascade. (A) RAW cells were transfected with control siRNA or with siRNA to mTOR (100 nM), and after 5 days, cells were washed, rested and infected with F. tularensis LVS for 90 min to assess bacterial invasion. Values are the mean ± SEM of 5 independent experiments, each done in triplicate; **p < 0.001; *p < 0.05 compared with infected control transfected with control siRNA. (B) RAW cells were transfected with control siRNA or with siRNA to mTOR (100 nM) or non-transfected, and after 5 days, cells were washed, rested, infected with F. tularensis LVS (MOI=20) for 0-90 min and then lysed. Total p70S6K and Akt, and phosphorylated p70S6K (Thr389 and Thr421/Ser424), 4E-BP1 (Ser65), S6 (Ser235/236 and Ser240/244), eI-F4E (Ser209) and Akt (Ser473) were assessed by Western analysis. Samples contained equal amount of protein. RAW cells transfected with control siRNA were used as negative controls. Gels are representative of three to five independent experiments. (TIF) [file pone.0083226.s002.tif]

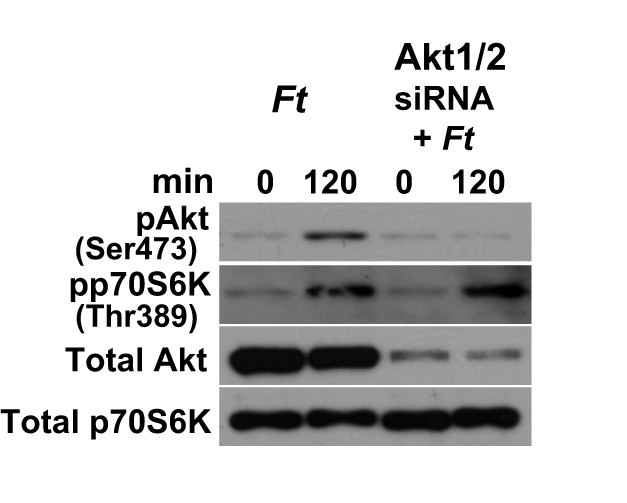

Supplement: Figure S3 — Phosphorylation of p70S6K in RAW cells transfected with siRNA to Akt1/2. RAW cells were transfected or not with siRNA to Akt1/2 (100 nM), and after 5 days, cells were washed, rested, infected with F. tularensis LVS (MOI=20) for 0 and 120 min and lysed. Total and phosphorylated p70S6K (Thr389) and Akt (Ser473) was assessed by Western analysis. Samples analyzed contained equal amount of protein. Unstimulated cells served as negative controls. Gels are representative of three to five independent experiments. (TIF) [file pone.0083226.s003.tif]

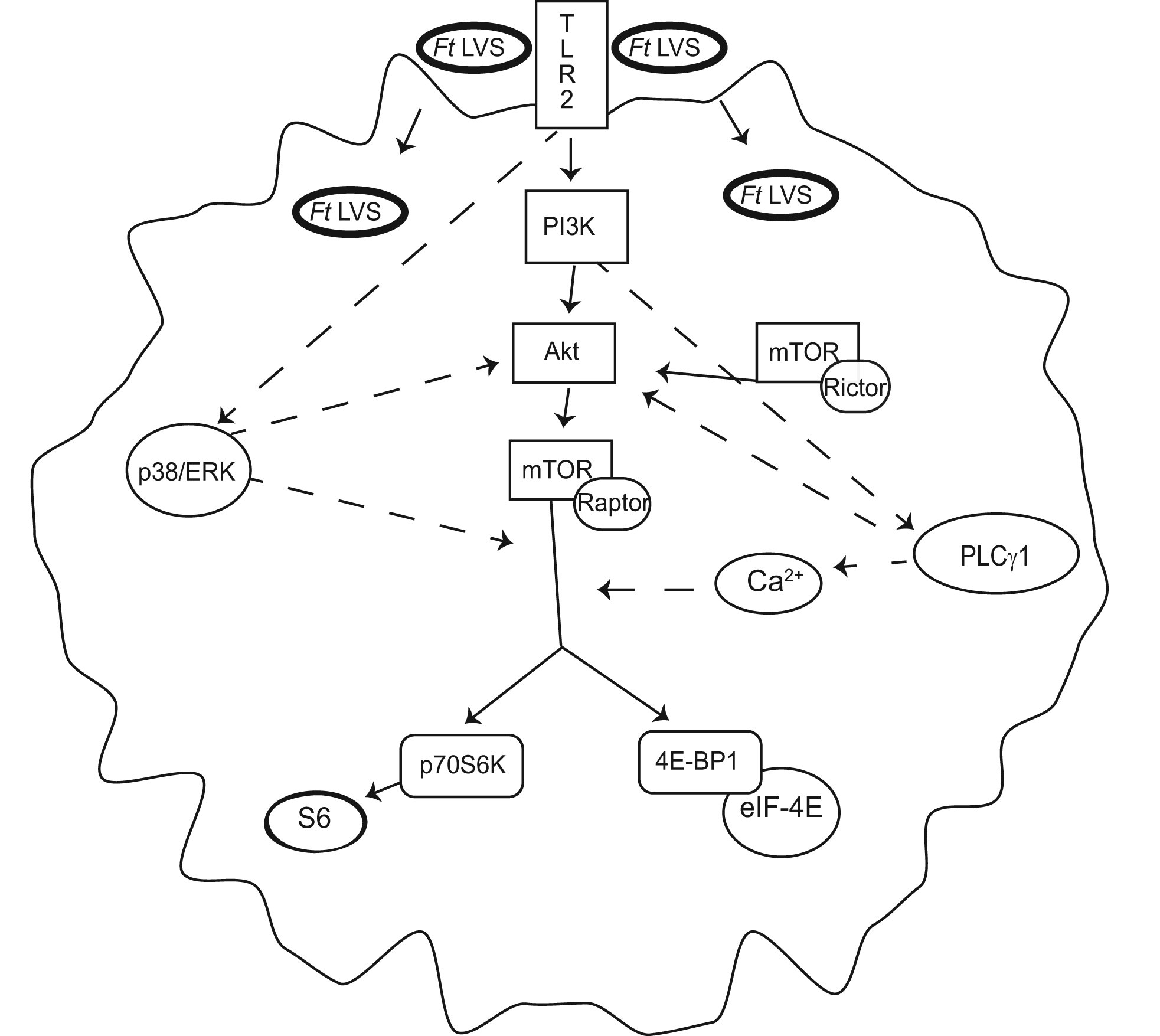

Supplement: Figure S4 — Proposed model of the signaling pathways involved in the phosphorylation of mTOR downstream effector molecules associated with F. tularensis invasion of primary macrophages via TLR2 signaling. (TIF) [file pone.0083226.s004.tif]
